# Physiological and subjective comfort evaluation under different airflow directions in a cooling environment

**DOI:** 10.1371/journal.pone.0249235

**Published:** 2021-04-14

**Authors:** Kaori Tamura, Sayaka Matsumoto, Yu Hsuan Tseng, Takayuki Kobayashi, Jun’ichi Miwa, Ken’ichi Miyazawa, Toyotaka Hirao, Soichiro Matsumoto, Seiji Hiramatsu, Hiroyuki Otake, Tsuyoshi Okamoto

**Affiliations:** 1 Faculty of Arts and Science, Kyushu University, Nishi-ku, Fukuoka, Japan; 2 Graduate School of Systems Life Sciences, Kyushu University, Nishi-ku, Fukuoka, Japan; 3 Research and Innovation Center, Mitsubishi Heavy Industries LTD., Nagoya, Aichi, Japan; 4 Mitsubishi Heavy Industries Thermal Systems LTD., Kiyosu, Aichi, Japan; University of Wuerzburg, GERMANY

## Abstract

Indoor comfort is influenced by airflow direction, but subjective evaluations can differ. This study evaluates the airflow comfort with subjective assessments and physiological measurements, including skin temperature, electroencephalograms, and electrocardiograms. Nineteen participants entered a test room at 20°C after staying in a room at 32°C for acclimation. They were exposed to *indirect* and *direct* airflow conditions to their faces and performed four tasks under each condition: resting, counting to 10 s following time alerts, counting to 10 s in mind, and mental calculation. Subjective assessments showed relatively higher thermal sensation and pleasantness under indirect airflow. The psychological time calculated from counting behaviors was longer under indirect airflow, indicating suppression of negative emotions. The face temperatures significantly declined during experiments under direct airflow. The beta and gamma bands of electroencephalograms were inhibited under the indirect condition, and these amplitudes were negatively correlated with pleasant feelings. Electrocardiogram parameters indicated that sympathetic nervous activity was predominant during counting, following alerts and mental calculation in indirect airflow. This study supports the comfort of indirect airflow based on reliable evidence.

## 1 Introduction

Airflow direction, along with velocity, can influence the comfort level in a cooling environment [1–4]. When evaluating thermal comfort, air movements are regarded as unwanted since high wind velocities decrease indoor comfort [1,3]. It has been reported that discomfort feelings varied depending on air direction [4]. Direct hitting of cool air reduces skin temperatures, leading to higher indoor discomfort. The satisfaction with the airflow direction will improve the quality of life and promote better work productivity in offices and schools.

The discomfort in indoor airflow velocities has long been researched, and the satisfaction varied depending on temperature, climate, and individuals [5]. Some studies have proposed the preferences of airflow movement in a cooling environment, especially in subtropical regions [6–9]. There are many subjective but few objective approaches for evaluating feelings of comfort. This imbalanced evaluation could be one possible cause that airflow effects on comfort remain controversial.

To assess indoor pleasantness, including airflow comfort, subjective assessments or rating scales are widely used. A common international scale, the Predicted Mean Vote (PMV) [10], was established to summarize and standardize these factors. However, the validity of PMV is controversial [11,12], especially in east Asian regions [13]. Differences between PMV and the actual mean vote in naturally ventilated and air-conditioned spaces have also been reported [14]. Since subjective evaluations can vary intra- and inter-individually according to physical and mental states, objective measurements should be added to the conventional approach.

Electroencephalography (EEG) is a strong candidate for the objective evaluation of airflow comfort. Some components of EEG signals are known to reflect mental states directly [15,16]. For example, beta and gamma amplitudes were found to be lower under a no-airflow condition than under a direct-airflow condition in a showhouse [17]. These amplitudes are useful indicators to evaluate comfort feelings with different airflow directions. While EEG signals reflect activities of the central nervous system, electrocardiogram (ECG) parameters can indicate the activity of the autonomic nervous system. The heart rate variability calculated from ECG signals provides two indicators: the high-frequency component (HF), which reflects vagus nerve activity related to parasympathetic nerves, and the ratio of low-frequency and high-frequency components (LF/HF) to estimate sympathetic activity [18].

The main purpose of this study is to evaluate how airflow direction influences the comfort feeling in a cooling environment produced by air-conditioners using subjective and objective measurements, including skin temperature, EEG, and ECG. The relationship between the subjective evaluation and physiological indicators, especially EEG, is also investigated. The physiological responses were measured under two airflow directions: direct and indirect cool air to the face. For EEG analysis, beta and gamma activities were extracted as per our previous report [17] as responses of the central nervous system. For ECG analysis, HF and LF/HF were calculated as responses of the autonomic nervous system. These physiological measurements were used to evaluate the comfort of airflow direction from different aspects with subjective assessments. The beta and gamma bands of EEG are discussed in various contexts. To demonstrate how EEG activities were induced in our experiments, we examine the relationship between the subjective assessments recorded in the same experiments.

## 2 Materials and methods

### 2.1 Participants

We recruited 19 university students to participate in our experiments (female, n = 8, male, n = 11; mean age ± SD = 21 ± 2.1 years). All participants were without neurological deficits based on self-reports. The experiments were approved by the Joint Ethics Committee of the Faculty of Arts and Science and Center for Health Sciences and Counseling at Kyushu University (201815, 201815–1). Written informed consent was obtained from each participant. All methods were performed per the approved guidelines.

### 2.2 Apparatus and protocol

Due to a schedule conflict between participants and the experimental room, the experiments were conducted in two periods, from 21 January 2019 to 1 February 2019, and from 10 May 2019 to 23 May 2019 in the same experimental room at Mitsubishi Heavy Industries LTD., Aichi, Japan. The data measured in the two periods were pooled. Environmental parameters, including room temperature, wind direction, and wind velocity, were monitored and controlled simultaneously from a control room outside the experimental room.

**Fig 1:**
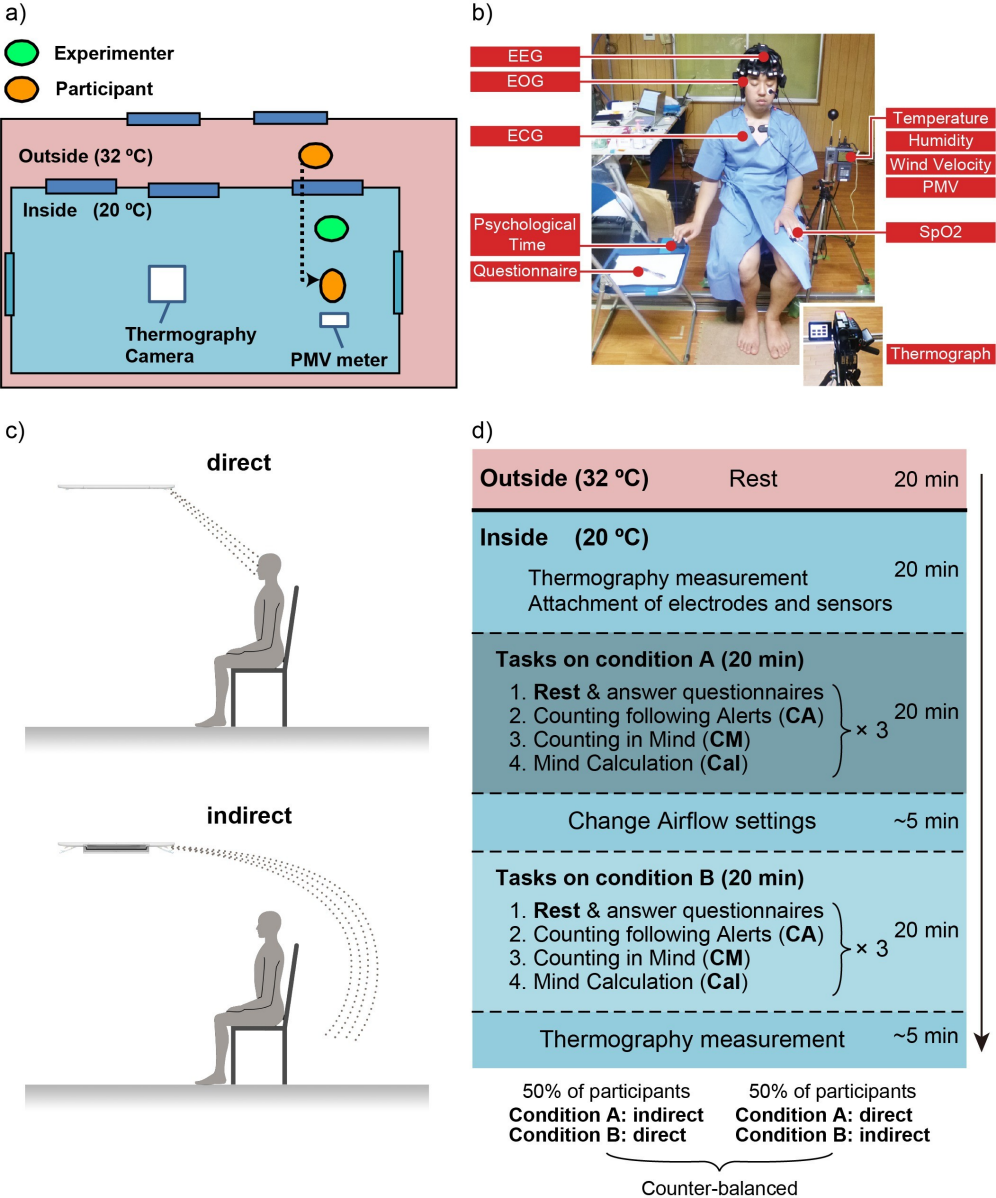
Experimental environment, settings, and procedure. a) Experimental environment. b) Sensor locations of the physical measurements and PMV meter. The thermography camera was located in front of the participants. c) Airflow directions of the two conditions, direct and indirect. d) Experimental procedure. The order of airflow settings was pseudo-randomized across participants. We obtained permission from the participants for printing the photographs of their faces.

The indoor condition was controlled using a commercially available air-conditioner with special panels (Draft Prevention Panel, Mitsubishi Heavy Industries Thermal Systems LTD., Tokyo, Japan) to avoid the direct impact of air from the indoor unit. Two different airflow conditions, see Fig 1, were set as follows: under direct airflow, the air from the air-conditioner was set to hit the participants’ faces; under indirect airflow, this was prevented by the control panels (Fig 1C). Staff stayed in the control room to monitor the physical parameters and to control the air-conditioner.

To simulate the outside temperature, during the summer in Japan, each participant was asked to stay in a waiting room with the temperature set to 32°C for 20 min. This acclimation to hot temperature counteracted the effect of variations in outside temperature during the experiment period. The participant then moved to the experimental room, where the room temperature was set to 20°C. 20 min after the participant had entered the experimental room, physiological measurements and the experiment were started (Fig 1D). The order of airflow conditions was counterbalanced for the participants. The air-conditioner was controlled before each measurement of a condition. Tasks the participants had to fulfill during the experiment consisted of four sessions: rest, time counting following alerts (CA), time counting in mind (CM), and mental calculation (Cal). This sequence was repeated three times for the direct and indirect airflow conditions in a random order (Fig 1D). The sequence for one airflow condition took around 20 min. After one sequence, including three sessions, was finished, the next sequence started immediately.

The participants wore an EEG headset in the experimental room and performed tasks under the different airflow conditions (Fig 1A). Each participant sat in a chair placed at the designated position near an experimenter. A PMV-meter was placed close to the participant, and a thermography camera was placed in the front (Fig 1A and 1B, see details in sections 2.3 and 2.4). EEG and ECG electrodes were attached to the participant immediately after entering the experimental room. The participants were asked to wear the same kind of hospital gown to standardize the clothing. The blood oxygen saturation (SpO_2_) was measured by a finger pulse oximeter to confirm safe oxygen levels of more than 95%. (Fig 1B). When we observed oxygen levels higher than the safety levels during the experiments, the results of SpO_2_ were not reported in the Results section.

In the rest session, the participants were instructed to keep their eyes closed for 1 min. After resting, each participant answered the questionnaire about thermal sensation (1: very cold, 7: very hot), pleasantness (0: very unpleasant, 100: very pleasant), fatigue (0: best condition and no fatigue, 100: worst condition and cannot do anything with extreme fatigue), sleepiness (1: fully awake, 9: very sleepy), and anxiousness (0: not feel anxious at all, 100: feel extremely anxious). The thermal sensation and sleepiness were assessed with 7 and 9 step discrete scales, respectively, and others with horizontal visual analog scales with 10-cm lines.

The CA and CM sessions were designed to measure psychological time. In the CA session, each participant was required to press a button following time alerts every 10 s for 60 s. That is, they pressed the button six times in a session. In the CM session, the participants were asked to estimate 10 s as accurately as possible and press the button after counting silently for 10 s; this task was repeated six times (~ 60 s). The measured psychological time in the CA and CM sessions were analyzed as described in section 2.6.

In the Cal session, each participant was asked to consecutively subtract 13 from a four-digit number (e.g., 1012−13 = 999, 999−13 = …) as quickly as possible for 60 s. The four-digit number was randomly assigned, ranging from 1000 to 1012 for each participant.

### 2.3 Wind velocity measurement

**Fig 2:**
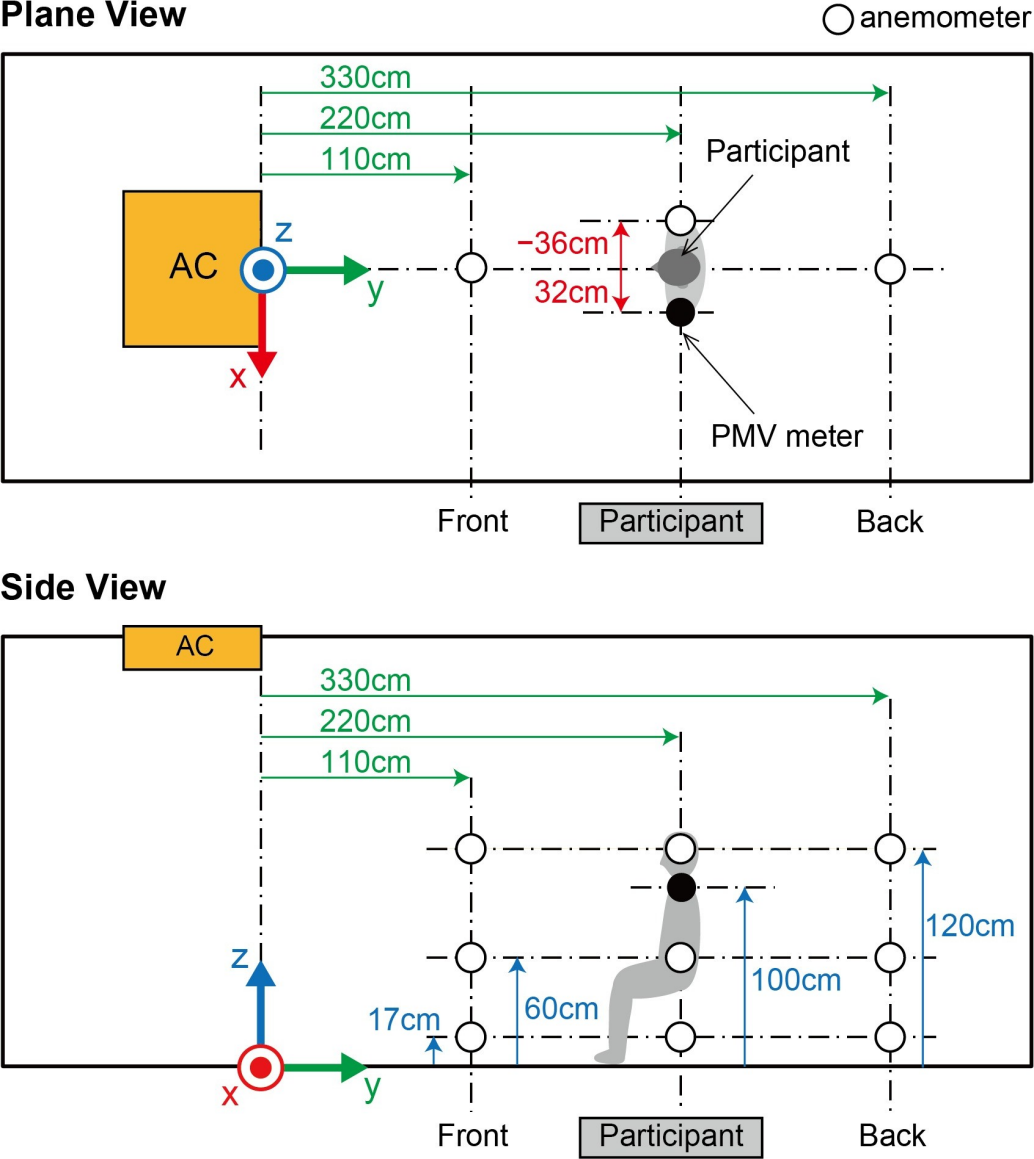
Measurement settings of wind velocity and direction. White circles indicate the nine locations where the wind velocity and direction were measured using an anemometer. The nine locations were combinations of the three points of the front-back direction and the three heights from the floor. Gray silhouette indicates the sitting position of a participant, and the black circle indicates the location of the PMV meter. AC indicates the location of the air conditioner.

To confirm the environment for physiological measurements of the participants, wind velocity and direction were measured using a multi-channel anemometer (Multi-channel anemometer 1550 series, KANOMAX JAPAN INC. Osaka, Japan) and probe cables (MODEL 1504 probe cable velocity channel, KANOMAX JAPAN INC. Osaka, Japan). The anemometer was set up at nine points (Fig 2). The minimum distance between the floor and anemometer was 17 cm due to the anemometer’s size. The measurement was controlled by an accessory software (MODEL S620-00 Ver 1.46, KANOMAX JAPAN INC. Osaka, Japan). The wind velocity and direction were measured for 1 min at a sampling rate of 10 Hz after waiting for a few minutes for the machine to stabilize. The sampled wind velocity data in each direction were averaged for the 1-min measurements.

### 2.4 Physical measurement and analysis

During the experiments, room temperature, relative humidity, and wind velocities were measured by a PMV meter (AM-101, Kyoto Electronics Manufacturing Co. LTD., Japan), placed close to the participant. For analyzing time-series changes, each parameter was segmented and averaged across three repetitions under each airflow condition, and two-way ANOVA (Airflow conditions × three repetitions) performed on the averaged data. The statistical analyses were conducted using JMP® 14SW (SAS Institute Inc., Cary, NC, USA).

### 2.5 Thermography measurement and analysis

Thermography data, to assess skin temperature, were measured before and after each condition (InfReC R550, NIPPON AVIONICS Co. LTD., Tokyo, Japan). In each thermographic image, the face area (178 × 140 pixels) was determined and cropped. The thermal data were averaged within the area, and two-way ANOVA was conducted (airflow conditions × before/after the measurement) for within-subject analysis.

### 2.6 Subjective assessments

The responses to the questionnaire, measured after resting, were analyzed using a two-way ANOVA for within-subject variance (airflow conditions × three repetitions). On finding an interaction effect, we analyzed the simple-main effect of the conditions separated by repetitions for multiple comparisons.

### 2.7 Psychological time

Psychological time has been discussed in relation to negative emotions, including boredom [19,20], which will lead to overestimating time passages [21]. In this study, we measured psychological time as an indicator of psychological stress caused by airflows.

The psychological time is defined as the difference between the mean response durations of the CM and CA sessions. A positive psychological time indicates that the individual perceived a slow pace of time. Their response durations after counting for 10 s were averaged at each 60 s, that is, there were three averaged data for each participant and each condition. The mean durations in CM were subtracted from the corresponding CA durations. A two-way ANOVA was then conducted for within-subject analysis (Airflow conditions × three repetitions). We did not remove any data related to the psychological time for the analysis. Each participant responded 6 times in each block, that is, counted for 10 s 18 times in an airflow condition. We did not remove any outlier from the response durations.

### 2.8 EEG and ECG

We recorded EEGs across 19 channels per the international 10–20 system [22] using dry active electrodes, CGX Flex, and Drypad sensors with a wireless headset (Quick-20, CGX, San Diego, U.S.A.). The reference electrode was placed on A1, as defined by the above system, and an additional electrode was placed on A2 for further analyses. For removing artifacts, the electrooculogram (EOG) was measured using AIM Generation 2 (CGX, San Diego, U.S.A.) by adding sensors to the headset. Bipolar horizontal and vertical EOGs were recorded by two additional electrodes placed below the left eye and lateral from the outer canthus of the right eye with a reference electrode placed on the forehead. The EEG and EOG were amplified and digitalized at a sampling rate of 500 Hz.

The measured EEG data were re-referenced to the averages of A1 and A2 and separated by task sessions to reduce the artifacts from ECG efficiently. The EEG data during each session of 60 s were segmented and filtered with a notch-filter of 60 Hz. The data were then transformed using a discrete Fourier transform (DFT) with a rectangular window to obtain the DFT coefficients. DFT analysis was performed using the ‘fft.m’ function in MATLAB (MathWorks, Inc., Natick, USA). Amplitudes of the frequency bin calculated from the DFT coefficients were averaged within the following frequency bands: beta (14 ≤ *f* < 30 Hz), and gamma (30 ≤ *f* < 50 Hz), where *f* indicates the frequency. The segmented data were rejected when EOG data exceeded ± 80 μV for eliminating eye blinking artifacts. EEG time-series changes for each oscillation-band were assessed using two-way ANOVA for within-subject analysis (Airflow conditions × three repetitions) in each session for each electrode. On finding an interaction effect, we analyzed the simple-main effect of the conditions separated by the repetition.

In addition to EEG and EOG channels, we recorded ECG using AIM Generation 2 (CGX, San Diego, U.S.A.). ECG was measured by the Bipolar Limb Lead method, which used a positive electrode at the left arm, and a negative electrode at the right with a reference electrode. These data were sampled at 500 Hz.

ECG data during each session, measured for 60 s, were segmented, detrended, and smoothed by a moving window. R-R intervals of each data were calculated by finding R-peaks. Then, DFT was employed for R-R interval data to obtain heart rate variability. Using the DFT components from R-R interval data, we calculated the mean components for HF (0.15 ≤ *f* < 0.4 Hz) and LF (0.04 ≤ *f* < 0.15 Hz). LF/HF was used as an indicator of sympathetic modulation, and HF was an indicator of parasympathetic activity. For testing of HF and LF/HF, we conducted two-way ANOVA for within-subject analysis across the two airflow conditions and three repetitions by the four sessions. On finding an interaction effect, we analyzed the simple-main effect of the conditions separated by the repetitions.

## 3 Results

### 3.1 Wind velocity and direction

**Fig 3:**
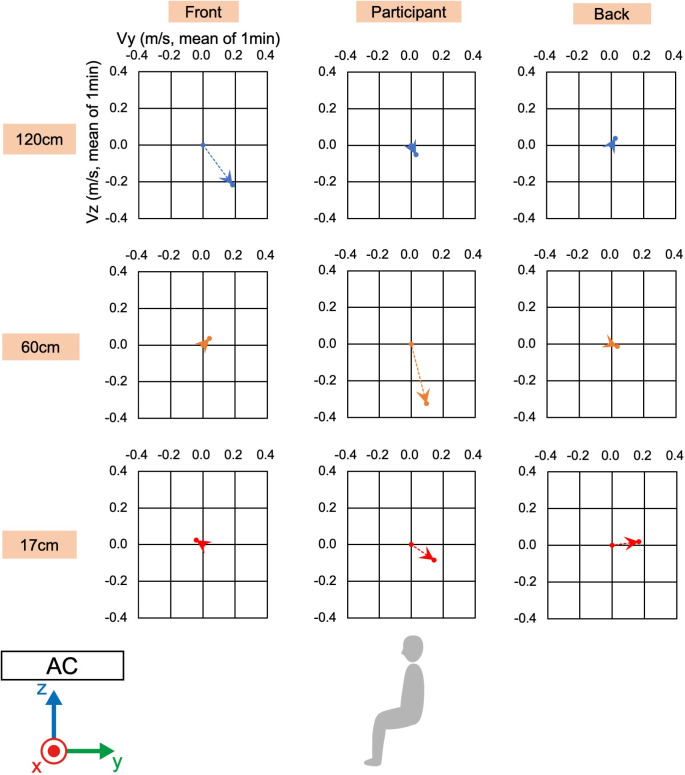
**Wind velocity and direction in Y-Z plane of direct condition.** Vy and Vz indicate the mean of the 1-min velocity in the Y or Z direction, respectively. Dotted lines indicate the vectors of the wind velocities.

**Fig 4:**
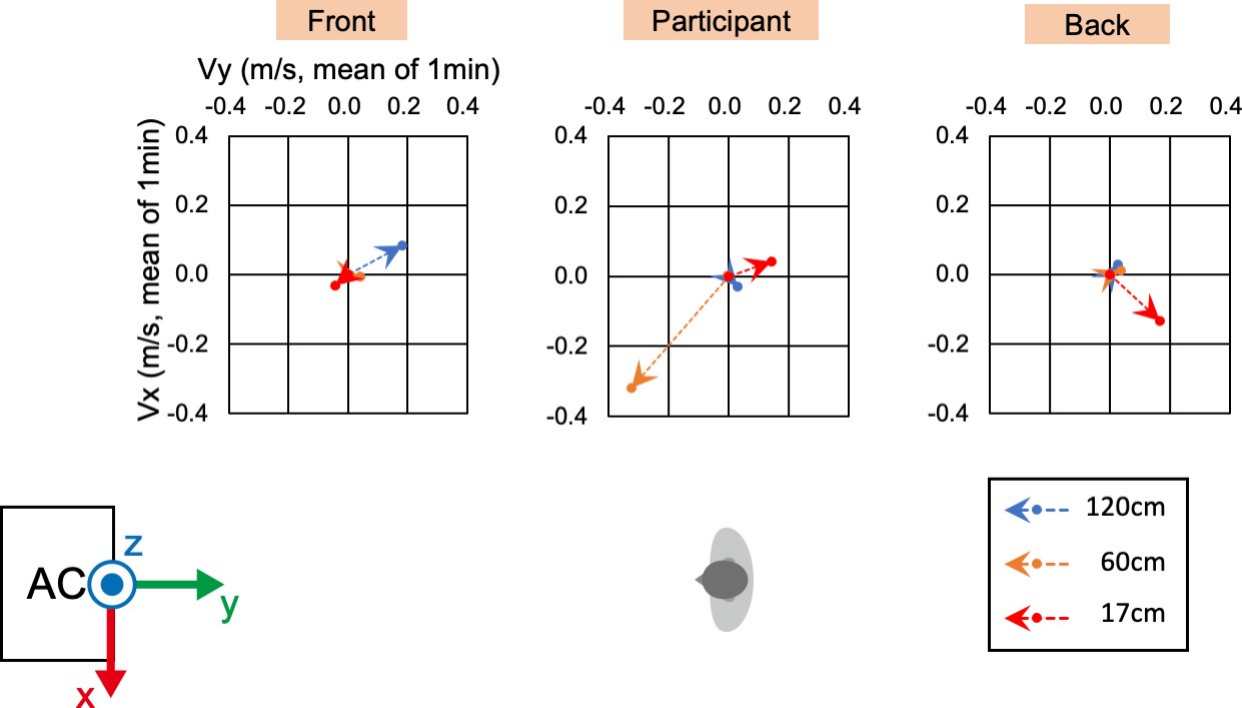
**Wind velocity and direction in the X-Y plane of the direct condition.** Vx and Vy indicate the mean of the 1-min velocity in the Y or Z direction, respectively. Dotted lines indicate the vectors of the wind velocities.

**Fig 5:**
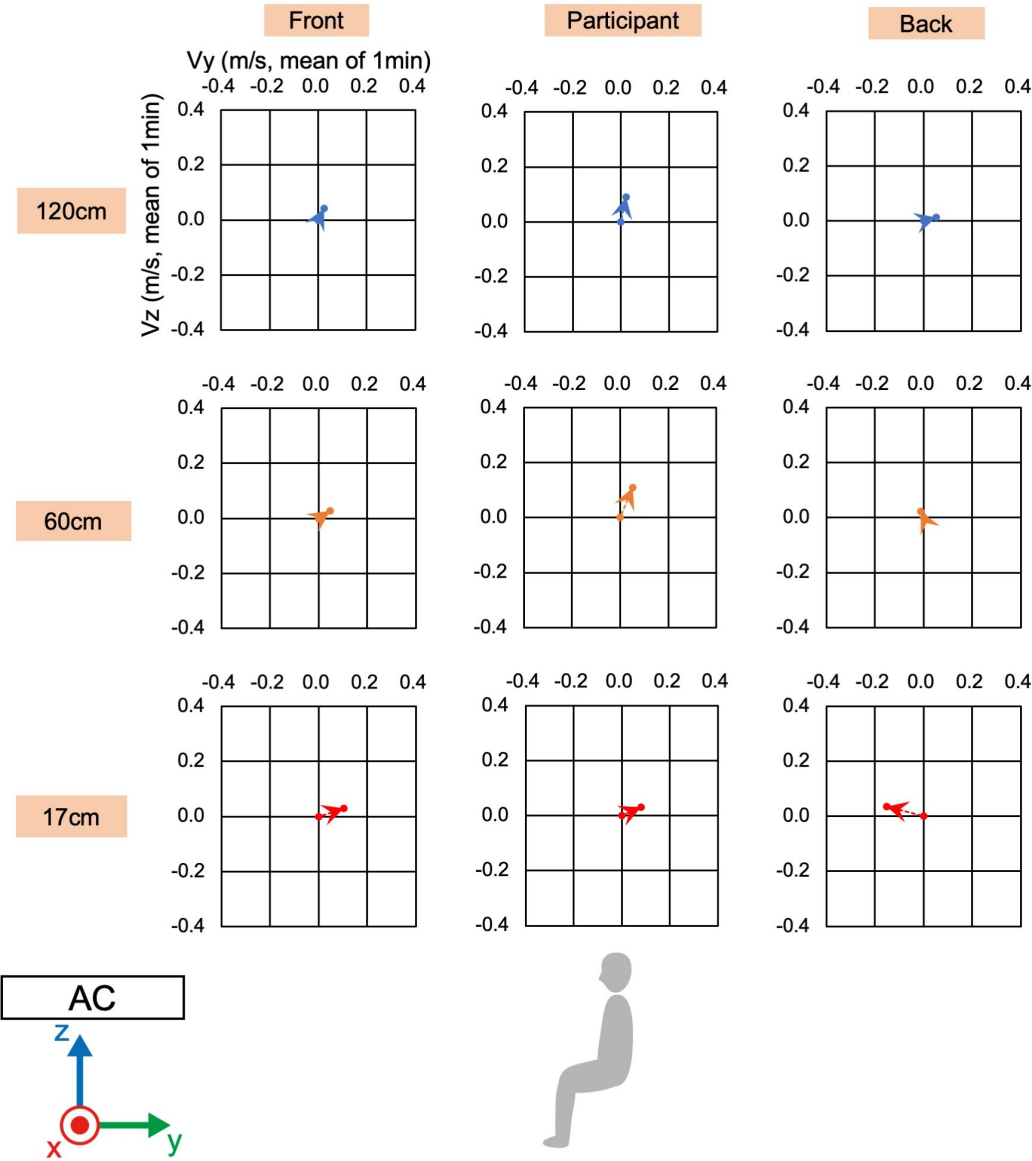
**Wind velocity and direction in the Y-Z plane of the indirect condition.** Vy and Vz indicate the mean of the 1-min velocity in the Y or Z direction, respectively. Dotted lines indicate the vectors of the wind velocities.

**Fig 6:**
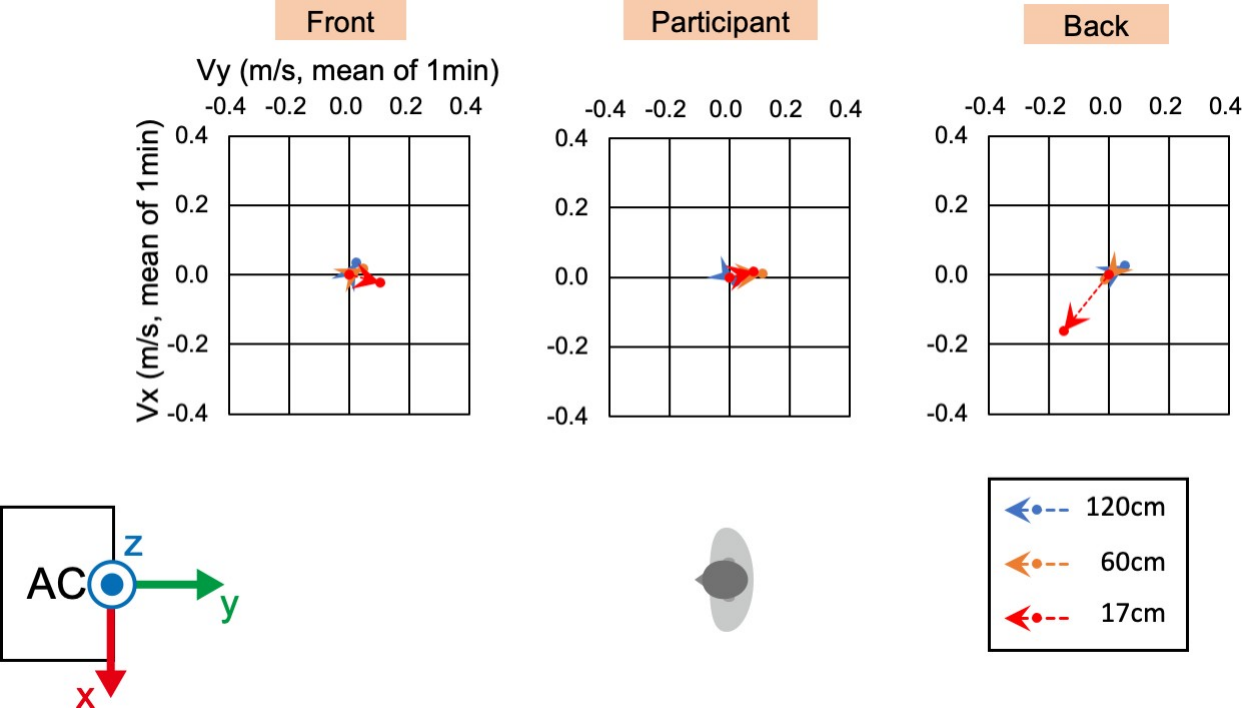
**Wind velocity and direction in the X-Y plane of the indirect condition.** Vx and Vy indicate the mean of the 1-min velocity in the Y or Z direction, respectively. Dotted lines indicate the vectors of the wind velocities.

Before the physical and physiological measurements, we confirmed the wind velocity and direction under the two conditions, i.e., direct and indirect airflow (Figs 2–6). Near the sitting site of the participant and at 60 cm height from the floor, the wind velocity under the direct condition was larger than under the indirect conditions, especially in the X-axis and Z-axis directions (Tables 1 and 2, Figs 3 and 5: 60 cm at the participant’s site). These data indicated that the wind direction and velocity were different between the conditions, especially around the sitting sites of the participants.

**Table 1:** Wind velocity under the direct condition.

Distance	Location	Wind Velocity [m/s]/mean of 1 min: X	Wind Velocity [m/s]/mean of 1 min: Y	Wind Velocity [m/s]/mean of 1 min: Z	Wind Velocity [m/s]/mean of 1 min: composite
120cm	Front	0.084	0.184	−0.217	0.338
120cm	Participant	−0.029	0.030	−0.050	0.126
120cm	Back	0.029	0.026	0.039	0.091
60cm	Front	−0.006	0.041	0.037	0.151
60cm	Participant	−0.319	0.094	−0.323	0.481
60cm	Back	0.012	0.036	−0.013	0.093
17cm	Front	−0.033	-0.042	0.025	0.137
17cm	Participant	0.043	0.143	−0.084	0.208
17cm	Back	−0.133	0.166	0.019	0.225

Mean of 1-min wind velocity [m/s] in each direction and composite vector length under the direct condition at each measurement position.

**Table 2:** Wind velocity under the indirect condition.

Distance	Location	Wind Velocity [m/s]/mean of 1 min: X	Wind Velocity [m/s]/mean of 1 min: Y	Wind Velocity [m/s]/mean of 1 min: Z	Wind Velocity [m/s]/mean of 1 min: composite
120cm	Front	0.034	0.024	0.043	0.087
120cm	Participant	−0.012	0.021	0.090	0.11
120cm	Back	0.025	0.051	0.014	0.093
60cm	Front	0.018	0.046	0.027	0.073
60cm	Participant	0.0090	0.051	0.11	0.14
60cm	Back	−0.013	−0.013	0.023	0.070
17cm	Front	−0.023	0.10	0.030	0.14
17cm	Participant	0.016	0.081	0.030	0.10
17cm	Back	−0.16	−0.15	0.035	0.23

Mean of 1-min wind velocity [m/s] in each direction and composite vector length under the indirect condition at each measurement position.

### 3.2 Physical measurements and common scales for thermal comfort

**Fig 7:**
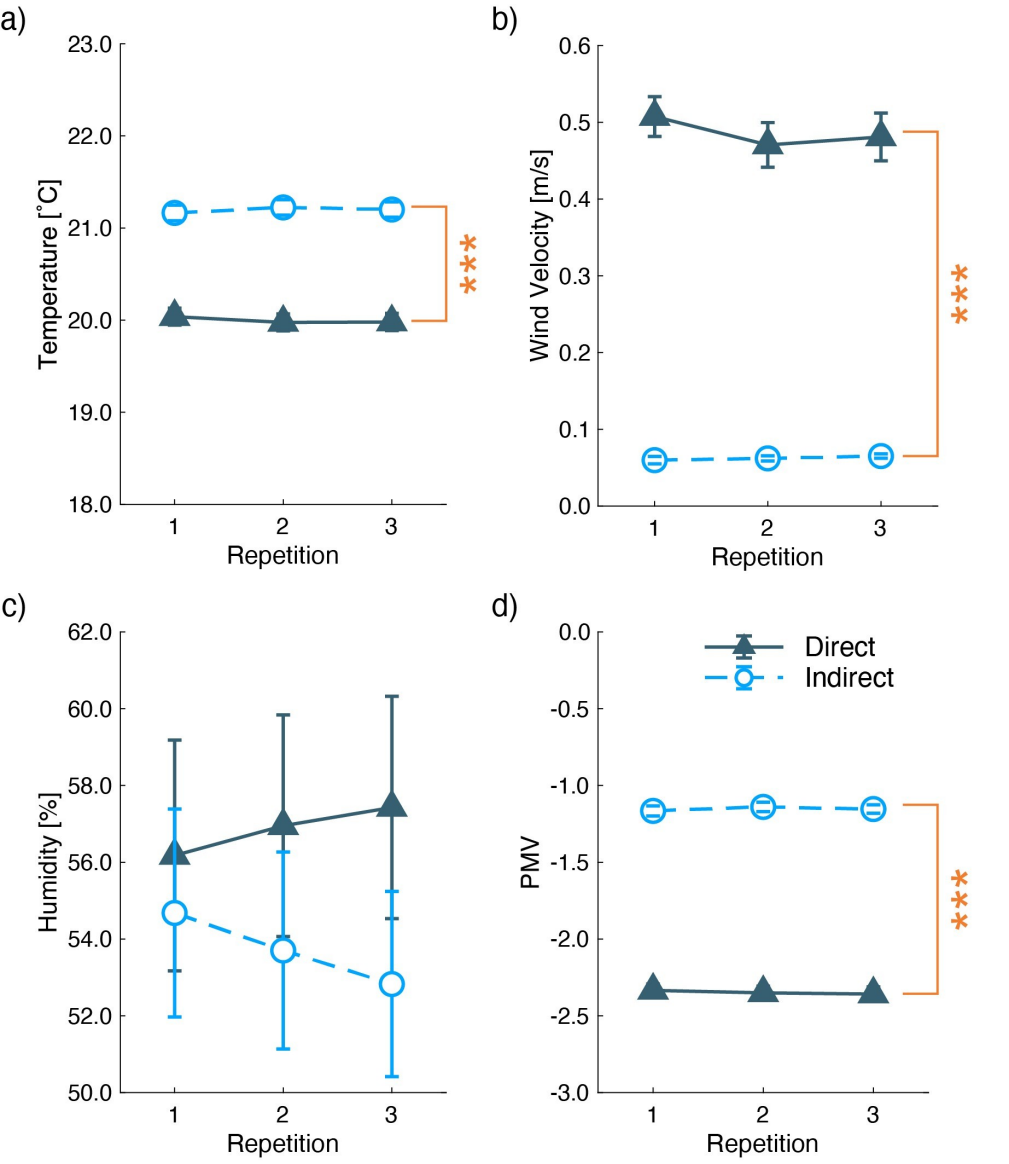
Mean and standard errors of a) room temperature, b) wind velocity, c) relative humidity, and d) predictive mean votes. The X-axis indicates three repetitions of the measurements. The U-shaped lines with asterisks show significant main effects of the airflow condition. (***: p < 0.001).

Room temperature, relative humidity, and air velocities near the participants were measured (Table 3, Fig 7). To show the time-related changes of these physical parameters, we conducted two-way ANOVA across the two airflow conditions and the three repetitions. The main effects of the airflow condition were shown in room temperature (*F*(1,104) = 39, *p* < 0.001) (Fig 7A) and wind velocities (*F*(1,104) = 682, *p* < 0.001) (Fig 7B). Relative humidity did not show any significant main effect in the airflow condition (*F*(1,104) = 1.935, *p* = 0.167) (Fig 7C). There was no significant main effect in the three repetitions (*F*(2,104) < 0.384, *p* > 0.682) and no interactions (*F*(2,104) < 0.547, *p* > 0.580) in room temperature, wind velocity, and relative humidity. These results indicate lower air velocity under the indirect airflow condition. Though the room temperature was kept at approximately 20°C in both conditions, there was a significant main effect due to wind velocity around the participants. The difference was, however, around 1.0°C, with small variances, and was not sufficient to conclude the failure of the indoor control parameters.

**Table 3:** Main effect of the two airflow conditions and p value from the ANOVA for physical parameters.

	Direct	Indirect	F (main effect of the conditions)	p
Room Temperature	20 ± 0.091°C	21 ± 0.082°C	39	<0.001
relative humidity	57 ± 2.9%	54 ± 2.6%	1.9	0.17
wind velocity	0.48 ± 0.029 m/s	0.062 ± 0.0032 m/s	682	<0.001

Mean ± SEM of the physical measurements across the three repetitions, and the main effects of the airflow conditions and their p-values obtained by two-way ANOVA (Airflow conditions × three repetitions).

To compare the thermal pleasantness and sensation in the experimental room, we compared PMV scales, which were used to predict the mean of thermal comfort evaluations as the sum of environmental variables, metabolic rate, and the level of clothing insulation [10]. Using two-way ANOVA (airflow conditions × three repetitions), the main effect of the airflow condition was found to be significant (*F*(1,104) = 1527, *p* < 0.001) (Fig 7D), indicating a significantly lower mean of predicted thermal comfort under the direct airflow.

### 3.3 Subjective assessments and behavioral results

**Fig 8:**
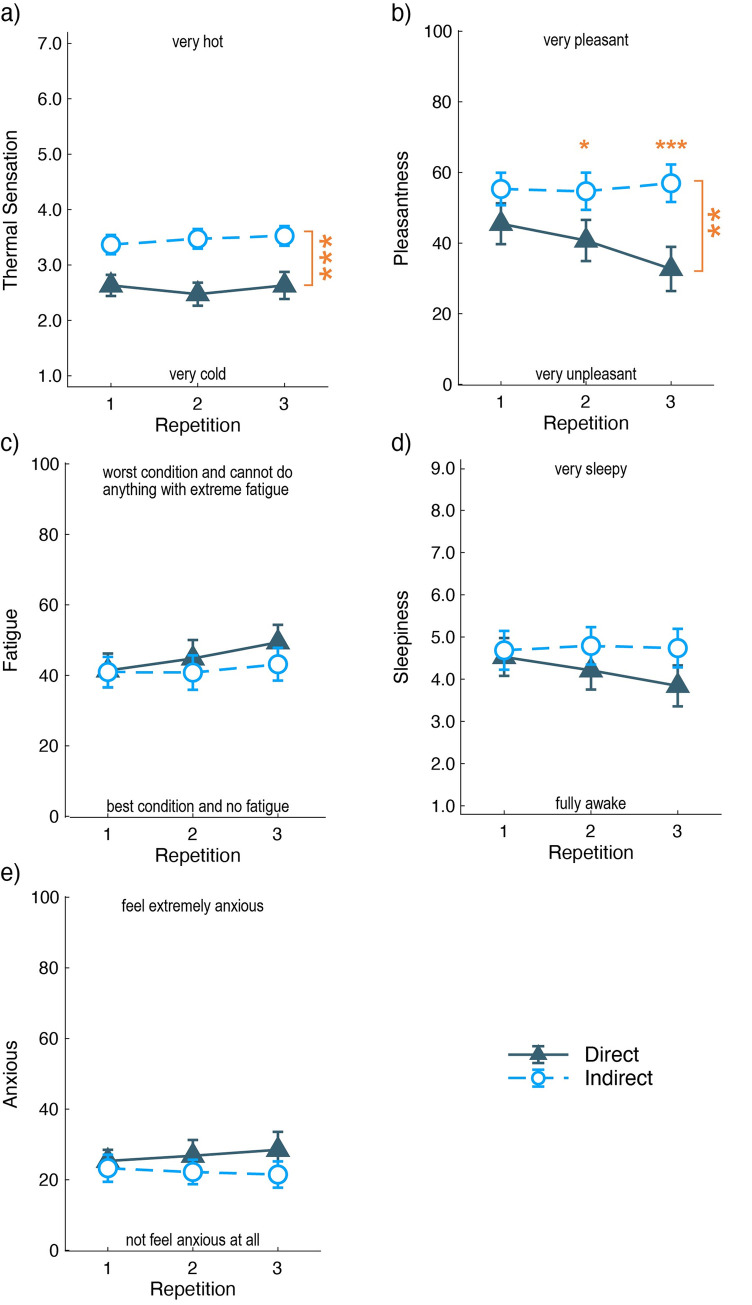
Mean and standard errors of the subjective assessments. a) Thermal sensation, b) pleasantness, c) fatigue level, d) sleepiness, and e) anxiousness. The X-axis indicates three repetitions of the measurements. The U-shaped lines with larger asterisks show significant main effects of the airflow condition obtained by two-way ANOVA. The smaller asterisk denotes a significant difference in the airflow condition at each repetition, obtained by multiple comparisons. (*: P < 0.05, **: P < 0.01, ***: P < 0.001).

Under each airflow condition, the participants repeated the questionnaires, involving thermal sensation, pleasantness, fatigue, sleepiness, and anxiety, thrice. The mean scores were indicated by the conditions and repetitions, as given in Fig 8.

The thermal sensation and pleasantness under the indirect airflow condition were evaluated higher than that under direct airflow. The two-way ANOVA (airflow conditions × three repetitions) for within-subject analysis showed a significant main effect of the air condition in thermal sensation and pleasantness (thermal sensation: *F*(1,18) = 25, *p* < 0.001, pleasantness: *F*(1,18) = 11, *p* = 0.004). There was no significant main effect of the repetition (*F*(2,36) = 1.194, *p* = 0.315) and interaction effect (*F*(2,2) = 1.500, *p* = 0.237) on the thermal sensation. However, there was a significant main effect of the repetitions (*F*(1,18) = 3.671 *p* = 0.035) and the interaction effect (*F*(2,36) = 4.891, *p* = 0.013) on pleasantness. Multiple comparisons show that pleasantness was significantly higher under indirect airflow for 2nd and 3rd measurements (2nd: *p* = 0.018, 3rd: *p* < 0.001). The results of thermal sensations indicate that the participants felt slightly cool and a higher pleasantness under the indirect airflow condition. Under the direct airflow condition, the pleasantness decreased with repetitions.

The fatigue levels showed a significant main effect of repetition (*F*(2,36) = 5.237, *p* = 0.0101) but no significant main effect of air condition (*F*(1,18) = 2.361, *p* = 0.142) and no interaction effect (*F*(2,36) = 0.98, *p* = 0.39). Sleepiness and anxiousness did not show any significant main effects and interactions (*F* < 2.914, *p* > 0.105).

**Fig 9:**
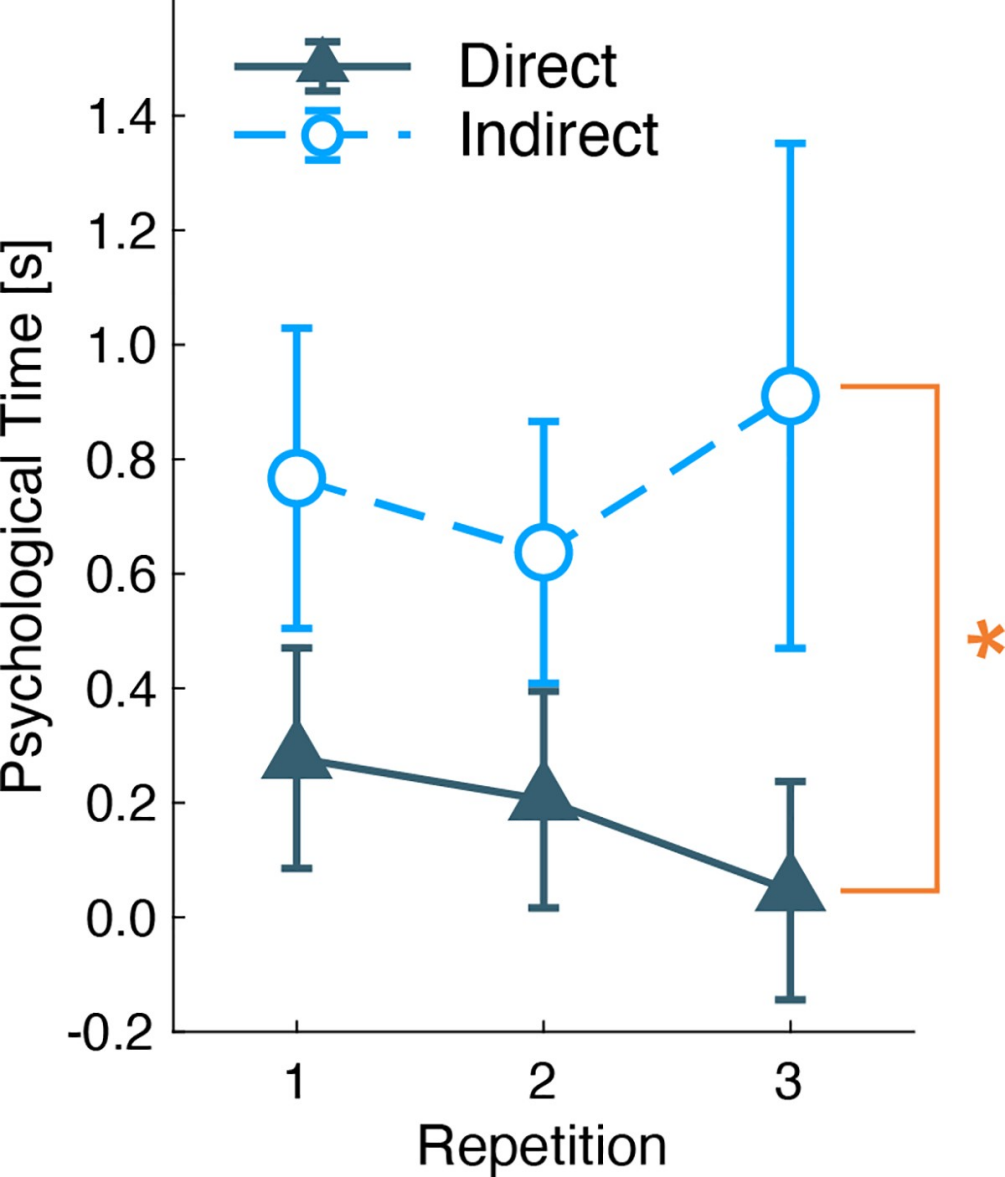
Mean and standard errors of the psychological time. The Y-axis indicates the psychological time, which is the difference between the mean response time in CM and CA. The X-axis indicates three repetitions of the measurements. The U-shaped line with an asterisk shows significant main effects of the airflow condition obtained by two-way ANOVA. (*: P < 0.05).

From the responses in the CA and CM sessions, the duration of psychological time under the indirect airflow condition was found to be longer across the three repetitions (Fig 9). The mean durations of the 10-s counts in the CA, CM, and the difference of CM and CA are summarized in Table 4. The psychological time calculated by subtraction of CA’s duration from CM’s duration can be used as indicator of the relative changes of performance speeds [23]. The reliability coefficients of psychological time were 0.934 and 0.777 under the direct and indirect condition, respectively (Table 4) [23,24]. The two-way ANOVA (airflow conditions × three repetitions) for within-subject analysis, showed a significant main effect of the airflow condition (*F*(1,18) = 6.270, *p* = 0.022) and did not show any significant main effect of the repetition (*F*(2,36) = 0.113, *p* = 0.894) and interaction effect (*F*(2,36) = 1.017, *p* = 0.372). This result supports the finding that the participants’ estimated psychological time is longer under the indirect than under the direct airflow condition.

**Table 4:** Reliability of psychological time.

	Number of Subjects	Mean Duration in CM [sec]	Mean Duration in CA [sec]	Psychological Time (CM—CA)	Spearman-Brown reliability coefficient
Direct	19	10.1	10.0	0.18	0.93
Indirect	19	10.6	10.0	0.77	0.78

Mean of CM duration, CA duration, psychological time (CM-CA), and reliability coefficients of the psychological time. The reliability coefficients were calculated by split-half method and the correlation coefficients were corrected by spearman-brown correction.

To evaluate the performance of mental calculation during the Cal session, the number of correct answers and the total mental calculations done by each participant were counted. A Chi-square test did not reject the null hypothesis that the correct rate was higher under one condition than under the other in each iteration (*χ*^2^ < 0.105, *p* > 0.745). The number of mental calculations was an indicator of the calculation speed, but the two-way ANOVA (airflow conditions × three repetitions) for within-subject analysis did not show any significant main effects of airflow conditions: *F*(1,18) = 0.156, *p* = 0.698, repetition: *F*(2,36) = 2.202, *p* = 0.125 and interaction effect (*F*(2,36) = 0.789, *p* = 0.462).

### 3.4 Skin temperature

**Fig 10:**
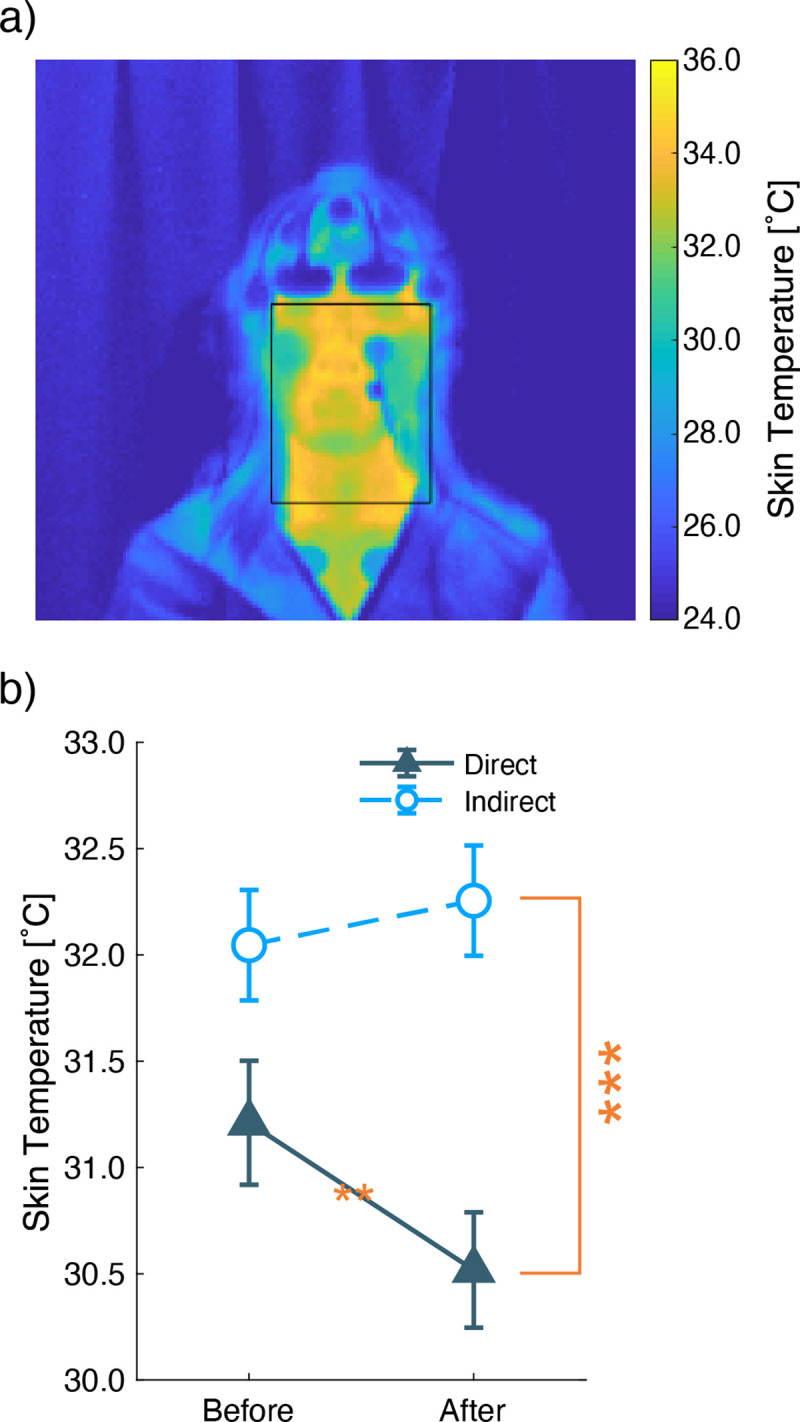
Face temperature measured by thermography images. a) An example of face area selection. b) Mean and standard errors of the face temperatures. The Y-axis shows the face temperature, which was calculated by averaging the thermography data of a face area. Thes X-axis shows two time points, before and after the experiment. The U-shaped line with larger asterisks shows significant main effects of the airflow condition obtained by two-way ANOVA. The smaller asterisk shows the significant difference before and after each airflow condition from multiple comparisons. (**: P < 0.01, ***: P < 0.001).

At the beginning and end of each experiment, the skin temperature of each participant’s face was measured by a thermographic camera. The measured face temperatures under the indirect airflow condition were higher than under the direct airflow (Fig 10). The two-way ANOVA (airflow conditions × before/after the condition) for within-subject analysis showed a significant main effect of the conditions (*F*(1,18) = 69, *p* < 0.001) and interaction effect (*F*(1,18) = 19, *p* < 0.001); however, there was no significant main effect of the measurement timings (*F*(1,18) = 1.855, *p* = 0.190) (Fig 10B). Under the direct airflow condition, the multiple comparison showed a significant decrease in temperatures before and after each experiment (*F*(1,29) = 11, *p* = 0.002), with no significant decrease under the indirect airflow condition (*F*(1,29) = 1.033, *p* = 0.318). These results indicate that the direct airflow carried the heat away from the faces during the experiments, though the face was in the indirect airflow.

### 3.5 EEG activities

In the EEG analysis, we focused on beta and gamma frequency bands since our previous study showed a relationship between these bands and airflow sensations [17]. The amplitudes were analyzed by each session and frequency band. The analytic electrodes were screened among 19 electrodes, whose criteria were the maximum and minimum values of the subtractions of the amplitudes measured under the direct condition from those under the indirect condition.

**Fig 11:**
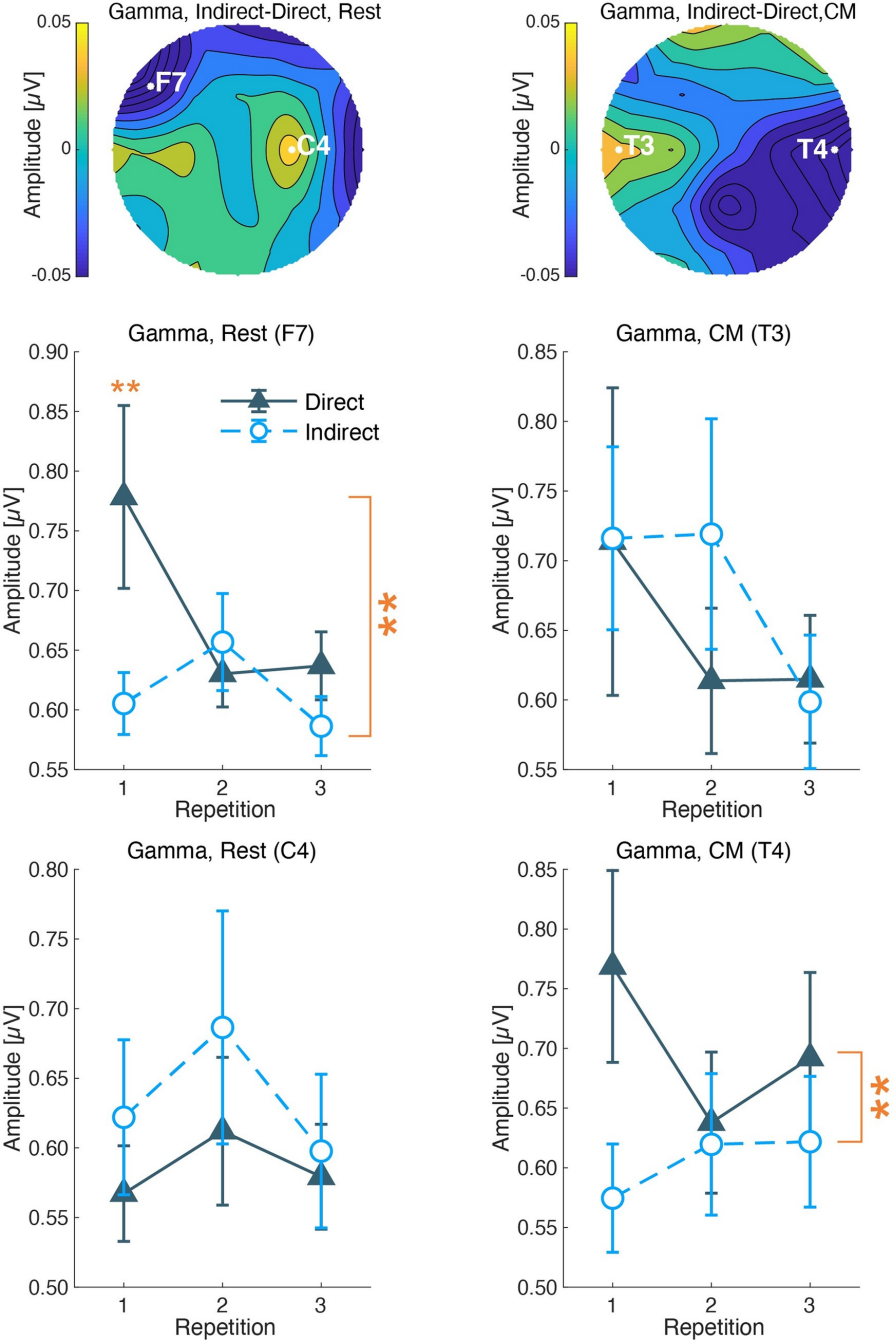
Gamma amplitudes during a) Rest and b) CM. The pop column shows the topographies of the mean difference between indirect and direct airflows. White letters and circles indicate the maximum and minimum electrodes of the subtracted amplitudes. The color bar shows subtracted amplitudes (μV). The middle column shows the mean and standard errors of the amplitudes at the maximum electrodes. The X-axis shows the repetition, and the Y-axis shows the μV of the EEG amplitudes. The bottom column shows the mean and standard errors at the minimum electrodes. The U-shaped lines with larger asterisks show significant main effects of the airflow condition obtained by two-way ANOVA. The smaller asterisk shows the significant difference in the airflow condition at each repetition, obtained by multiple comparisons. (**: P < 0.01).

In the rest session, the subtracted gamma amplitudes showed minimum value at F7 and maximum at C4 (Fig 11A). The two-way ANOVA at F7 indicated a significant main effect of the airflow condition (*F*(1,53) = 9.640, *p* = 0.003) and an interaction effect (*F*(2,36) = 3.605, *p* = 0.037). There was no significant main effect of the repetitions (*F*(2,36) = 2.826, *p* = 0.072). The multiple comparison showed a significant difference between the airflow conditions in the first measurement in the three repetitions (*F*(1,53) = 9.640, *p* = 0.003), but no significant difference in second and third measurements (*F*(1,53) < 0.821, *p* > 0.369). The amplitudes at C4 did not show any significant effects in the two-way ANOVA (*F* < 1.277, *p* > 0.291) (Fig 11A).

In the CM session, the subtracted gamma amplitudes showed a minimum value at T4 and the maximum at T3 (Fig 11B). The two-way ANOVA at T4 indicated a significant main effect of the airflow condition (*F*(1,52) = 7.279, *p* = 0.009), but not of repetition (*F*(2,36) = 0.656, *p* = 0.525) and the interaction effect (*F*(2,36) = 1.380, *p* = 0.265). The amplitudes at T3 did not show any significant main or interaction effects (*F* < 1.837, *p* > 0.174) (Fig 11B).

In the CA and Cal sessions, the gamma amplitudes did not show any significant main or interaction effects at the electrodes of maximum (Fp2 during CA, Fp1 during Cal) and minimum (Pz during CA, F7 during Cal) (*F* < 1.776, *p* > 0.189) (S1 Fig).

**Fig 12:**
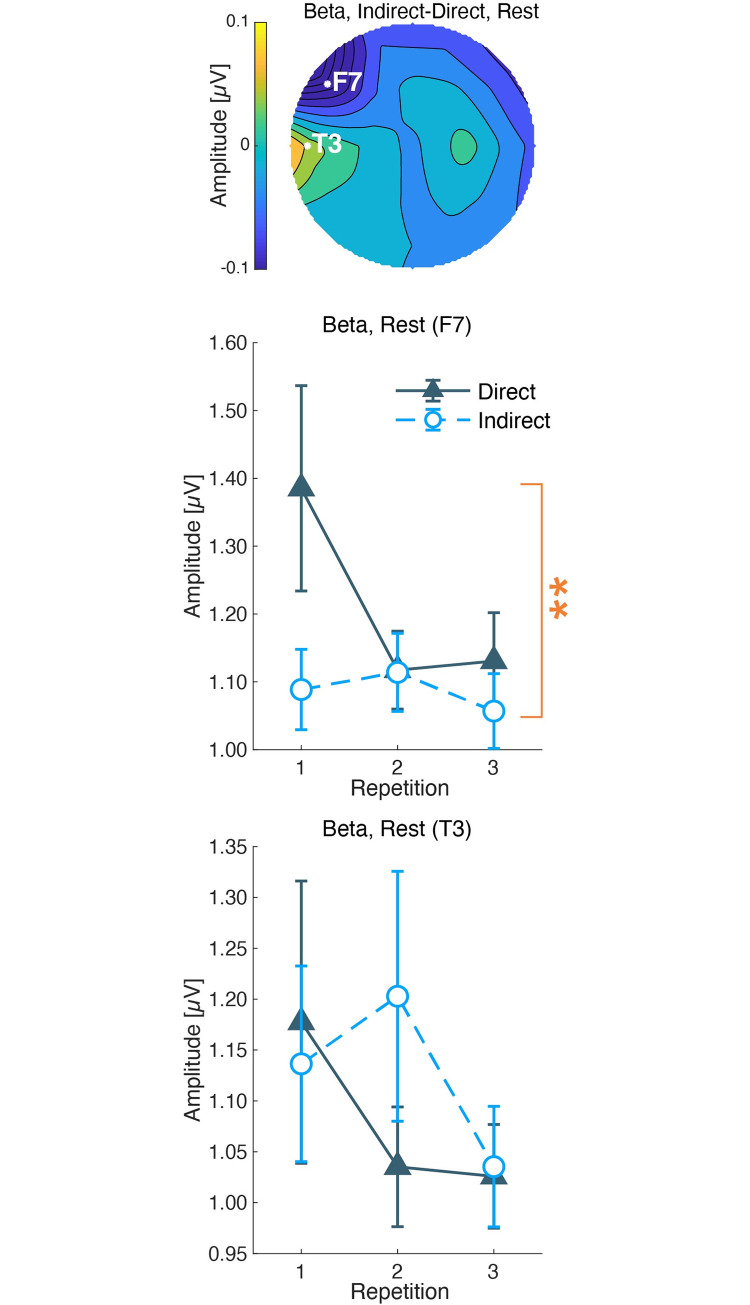
Beta amplitudes during rest. The top column shows the topographies of the mean difference between indirect and direct airflows. White letters and circles indicate the maximum and minimum electrodes of the subtracted amplitudes. The color bar shows the subtracted amplitudes (μV). The middle column shows the mean and standard errors of the amplitudes at the maximum electrodes. The X-axis shows the repetition, and the Y-axis shows the μV of the EEG amplitudes. The bottom column shows the mean and standard errors at the minimum electrodes. The U-shaped line with asterisks shows significant main effects of the airflow condition obtained by two-way ANOVA. (**: P < 0.01).

The beta amplitudes were analyzed in the same way as the gamma amplitudes (Fig 12). The minimum beta amplitudes in the rest session were at F7, and the maximum at T3. For the amplitudes at F7, the two-way ANOVA showed a significant main effect of the airflow condition (*F*(1,52) = 8.859, *p* = 0.004) and repetition (*F*(2,36) = 3.670, *p* = 0.035), while there was no interaction effect (*F*(2,36) = 2.705, *p* = 0.080). The multiple comparisons showed a significant difference in the first measurement (*F*(1,52) = 8.859, *p* = 0.004), but no significant differences in the second and third measurements (*F*(1,52 < 0.547, *p* > 0.463)). The two-way ANOVA at T3 did not show any significant main or interaction effects (*F* < 2.362, *p* > 0.109).

In the beta amplitudes of the CA session, the maximum site was the T3 electrode. The beta values showed a significant main effect of repetition (*F*(2,36) = 3.704, *p* = 0.034) but no significant main effect of the airflow or interaction effect (*F* < 0.555, *p* > 0.579). The minimum amplitude was shown at Pz, but there was no significant effect (*F* < 0.503, *p* > 0.609) (S2A Fig).

In the CM and Cal sessions, the amplitudes did not show any significant main or interaction effects at the electrodes of maximum (T3 during CM and Cal) and minimum (Pz during CM, and F7 during Cal) (*F* < 2.330, *p* > 0.112) (S2B and S2C Fig).

### 3.6 ECG

To investigate the airflow effects on the autonomic nervous system, parameters from ECG recordings were analyzed. HF reflects the parasympathetic activity, and LF reflects the modulation of sympathetic and parasympathetic activity [25]. LF/HF infers the sympathetic activity so that the parasympathetic power, to some extent, cancels out the HF decline [26].

**Fig 13:**
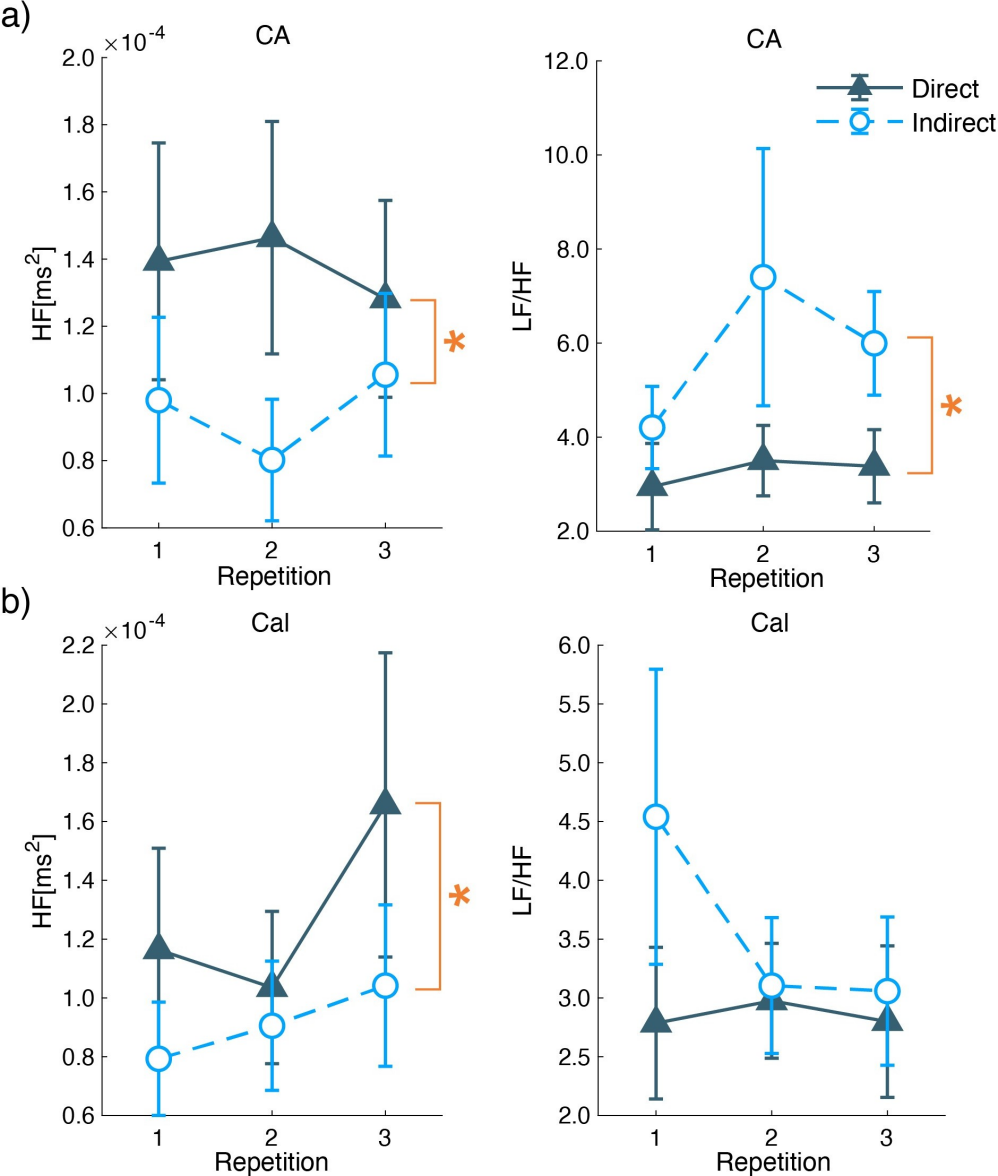
ECG parameters of HF and LF/HF during a) CA and b) Cal. The right row shows the mean and standard error of HF, and the left row shows that of LF/HF. The X-axis shows the repetition. The U-shaped lines with larger asterisks show significant main effects of the airflow condition obtained by two-way ANOVA. (*: P < 0.05).

During the CA session, HF under the indirect airflow condition was lower than that under direct airflow (Fig 13A). The two-way ANOVA indicated a significant main effect of the airflow condition (*F*(1,15) = 6.534, *p* = 0.022). LF/HF under the indirect airflow condition was higher, and there was a significant main effect of the airflow condition (*F*(1,15) = 4.875, *p* = 0.043) (Fig 13A). In HF and LF/HF tests, there was no significant main effect of the repetition (*F*(2,30) < 1.142, *p* > 0.333) and interaction effect (*F*(2,30) < 1.899, *p* > 0.183).

In the Cal session, HF under the indirect airflow condition was lower than that under the direct airflow condition (Fig 13B). The two-way ANOVA indicated a significant main effect of the airflow condition (*F*(1,15) = 6.417, *p* = 0.023). There was no significant main effect of the repetition and interaction effect (*F*(2,30) < 2.246, *p* > 0.124). LF/HF did not show any significant effects (*F* < 2.115, *p* > 0.138) of the air condition, repetition, and interaction (Fig 13B).

For the other sessions, the ECG parameters did not show any significant main and interaction effects (*F* < 3.532, *p* > 0.080) (S3A and S3B Fig).

### 3.7 Relationship between EEG and subjective assessments

The EEG activities, especially in the gamma band, have demonstrated many relations with various mental states or cognitive functions. To investigate the relevance of the observed EEG responses and indoor comfort, we conducted a correlation analysis with the subjective assessments and EEG amplitudes. In this analysis, the direct and indirect airflow conditions data were pooled to show the meaning of the EEG function, not to compare between the groups. The analytic frequency bands and electrodes were selected by the statistical significance of the two-way ANOVA (airflow conditions × three repetitions).

**Fig 14:**
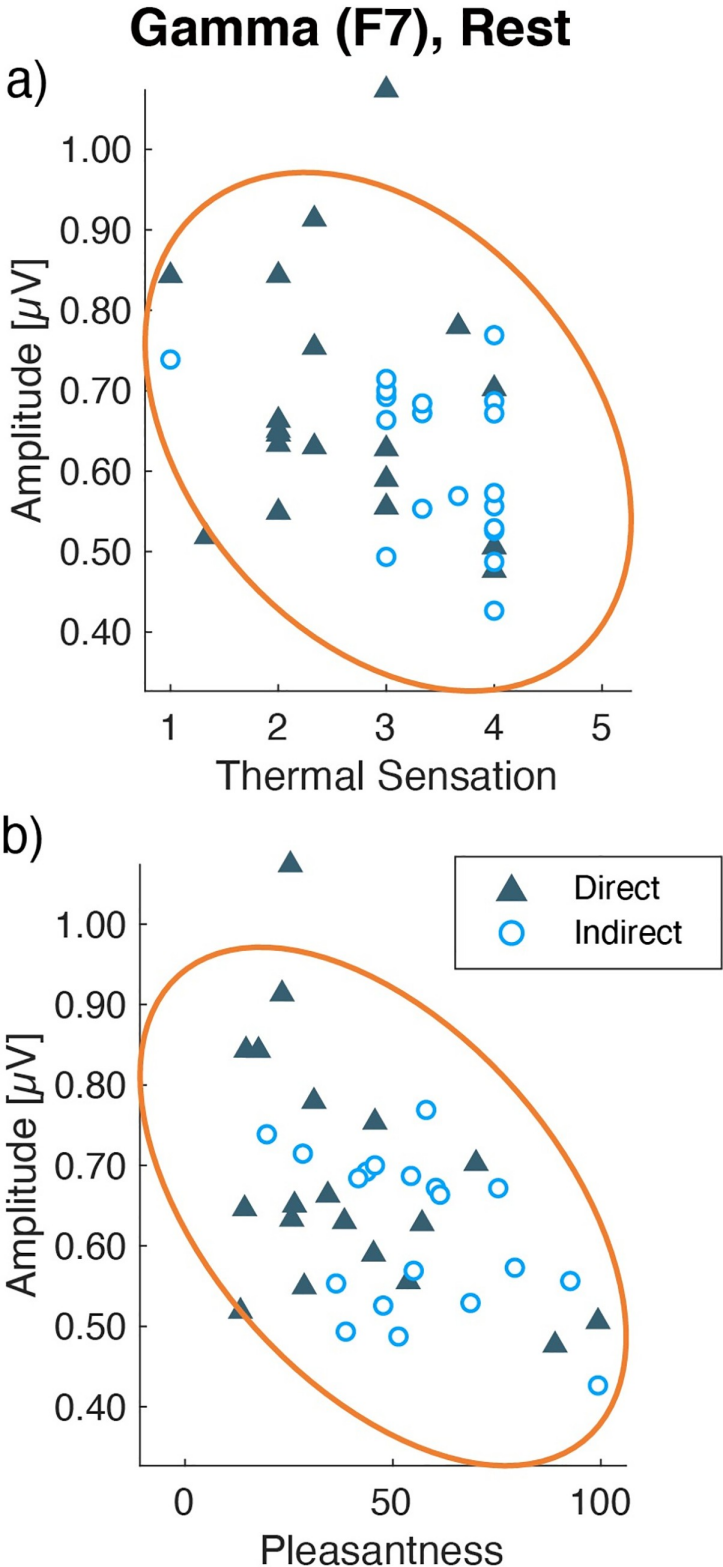
Scatter plots of each subjective assessment and gamma amplitudes at F7 during rest. Yellow lines show the 95% density ellipse. Each point indicates individual mean data across three repetitions.

The gamma amplitudes and subjective assessments were averaged across the repetition in each session and analyzed for correlations. In the rest session, gamma amplitudes at F7 were negatively correlated with thermal sensation (*r* = −0.341, *p* = 0.036), and pleasantness (*r* = −0.503, *p* = 0.001) (Fig 14), indicating that an increase in gamma at F7 could be related to unpleasantness during the rest session. With the other subjective assessments, there were no significant correlations with gamma amplitudes at F7 (|*r*| < 0.167 *p* > 0.031). In the CM session, the five subjective assessments at T4 did not show any significant correlations (*p* > 0.010). The gamma amplitude at T4 during the CM session did not show any significant correlation with each subjective assessment (|*r*| < 0.295, *p* > 0.072) (S4 Fig).

**Fig 15:**
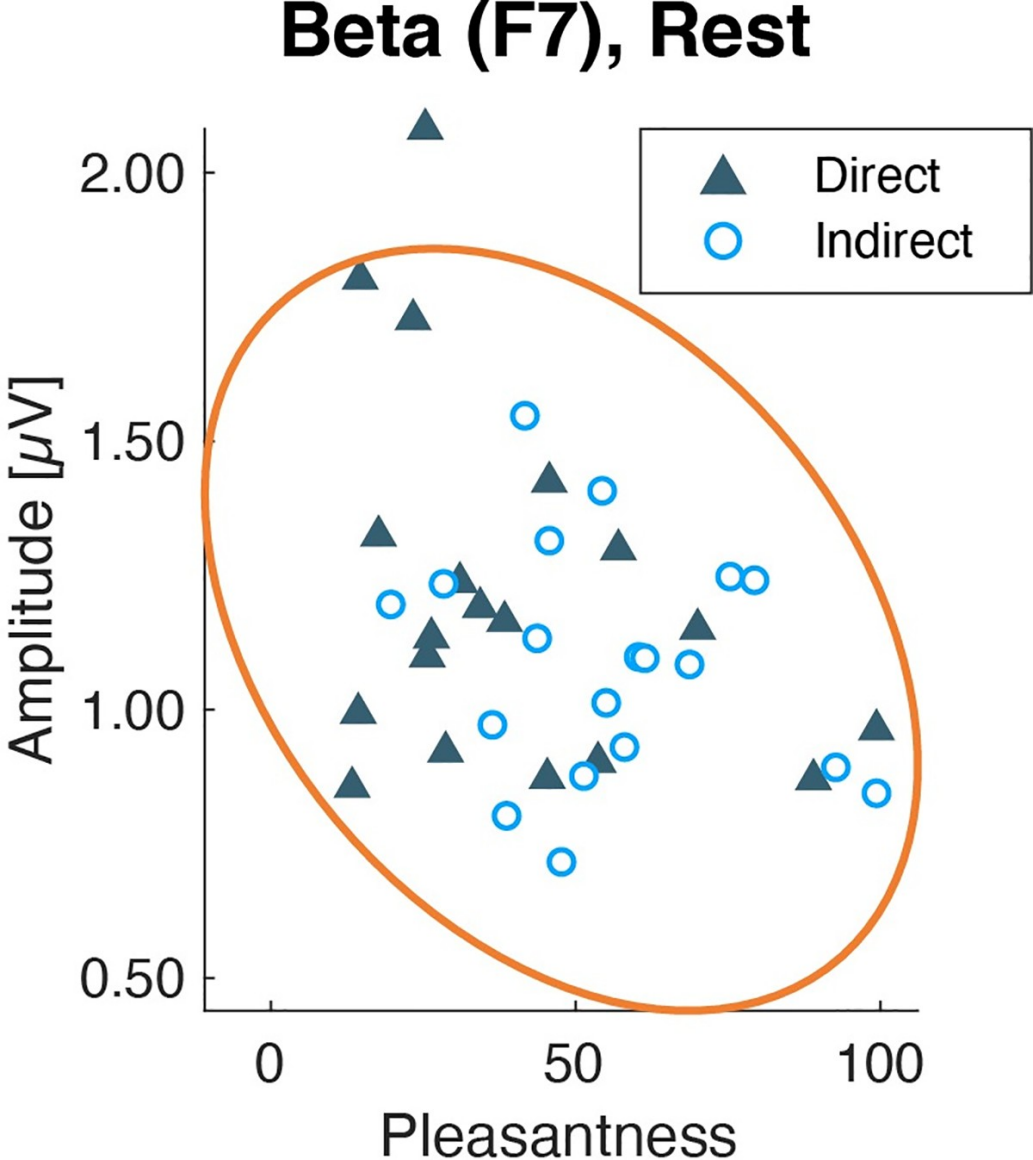
Scatter plots of each subjective assessment and beta amplitudes at F7 during rest. Yellow lines show the 95% density ellipse. Each point indicates individual mean data across three repetitions.

The beta amplitudes at F7 in the rest session were analyzed for correlation with subjective assessments (Fig 15). There was a significant negative correlation between pleasantness and beta amplitudes (*r* = −0.356, *p* = 0.028), while there was no significant correlation with other subjective assessments (|*r*| < 0.235, *p* > 0.155) (S5 Fig).

## 4 Discussion

In this study, we aimed to evaluate the effects of the environment, which were induced by different airflow directions, on indoor comfort with subjective assessments and physiological measurements. As expected, we confirmed that the subjective assessments and some physical measurements indicate higher pleasantness under the environment with indirect airflow. Furthermore, EEG and ECG activities showed different patterns between the airflow conditions. The gamma and beta amplitudes correlated with some subjective evaluations, including pleasantness and thermal sensation. The parameters of ECG, such as HF and LF/HF, were discussed in line with sympathetic modulation and parasympathetic activity, respectively. The different airflow environment could induce these differences in physiological responses, which are reflected in the airflow comfort levels.

From the physical measurements, we confirm that the indirect airflow maintained a mild room temperature and low wind velocity around the participants. The room temperature under the direct airflow condition was significantly lower, probably because of the higher wind velocity. A significant main effect occurred because there was a small variance in the room temperature due to the tight control of the environment. The difference in the room temperature was not large enough to influence the mental state or subjective pleasantness. The differences in indoor parameters modulated the dissociation of PMV between the airflow conditions. Furthermore, the different airflow directions influenced face temperature changes. The airflow hitting the face lowered the face temperatures under the direct condition, while these temperatures were maintained under the indirect condition. The large changes in face temperature may be one of the main factors that contributed to the pleasantness ratings discussed as follows.

The subjective assessments indicate that the indoor environment with indirect airflow was more pleasant than that with direct airflow. The room temperature around the participants was significantly varied between the airflow conditions, although the air-conditioner was set to the same temperature (20°C). The differences in subjective assessments and skin temperatures reflected the airflow conditions and room temperature, respectively. The participants evaluated that the environment under the indirect airflow condition was more pleasant and less cold than that under the direct airflow condition. This evaluation conformed with the scores of PMV. The mean pleasantness level was kept during the three repetitions under the indirect airflow condition, while it decreased with the repetitions under the direct airflow condition. Based on these results, we suggest that indirect airflow can maintain comfort feelings, while direct airflow reduces comfort feelings over time. The gradual decrease in face temperatures was similar to the time change of pleasantness ratings under the direct airflow condition, while the room temperatures did not show any time-series change. The lowering of face temperatures may contribute to the time-series dissociation of the pleasantness ratings between the airflow directions. Thus, skin temperature change can influence airflow comfort.

The duration of the psychological time was longer under the indirect airflow condition, and the results of the mental states supported the higher comfort under the indirect airflow condition. Our previous study has shown that the psychological time is longer in a no-airflow environment than under direct airflow exposure [17]. A longer psychological time can indicate a faster passage of the mental time; that is, the participants perceive a faster pace of mental time. In contrast, a shorter psychological time indicates a slower passage of the mental time, which suggests the relationship with negative mental states, including boredom [21] and anxiousness in threatening situations [19,20]. In line with these reports, indirect airflow exposure could inhibit a negative mental state and result in a longer psychological time.

The EEG responses also supported the pleasantness of the indirect airflow. The beta and gamma amplitudes of EEG activity showed lower amplitudes under the indirect airflow condition than under the direct airflow condition. We have reported that EEGs of gamma and beta amplitudes were lower in a no-airflow environment than those under a directed airflow from an air-conditioner [17]. From the present results and the previous study, we confirmed that a higher gamma activity is related to the airflow sensation. Regarding the rest session, we found a significant negative correlation between pleasantness or thermal sensation with the gamma amplitudes at the left frontal site during resting. The relationship between gamma activity and cold thermal sensation has been reported at the frontotemporal area by cortical measurements [27]. The observed differences in gamma amplitudes may be involved in cold thermal sensation induced by air direction. Additionally, in line with previous reports [17], we suggest that the gamma activity at the left frontal site was related to thermal unpleasantness under the different airflows during the calm and resting state. In contrast, the gamma amplitude during the CM session did not show a significant correlation with subjective assessments, although it showed a significant main effect for the airflow condition. The larger individual variability of mental states of the above result may be derived from the necessity of a collaboration of several brain functions to perform the CM task. During the CM session, the participants were asked to estimate 10 s and to press a button immediately. This instruction demanded to orchestrate attentional control, time perception, and estimation, and motor control to press a button, which may have caused different stress levels in individuals. The mental state during the CM session involved many aspects of cognitive function other than the measured subjective assessments. Further measurements of subjective stress will help to investigate the gamma activity during the CM session in future studies. The beta activity at the left frontal site showed significantly lower amplitudes under the indirect airflow condition and was negatively correlated with pleasantness during the rest session. This result was supported by a previous report, which showed the association between beta decrease and pleasantness at room temperature [28]. The authors compared moderate and warm environments and found beta amplitudes to be lower in moderate environments. Furthermore, a related study indicates that a stressful indoor environment induces higher beta activity at temporal sites [29]. Hence, we conclude that the beta decrease was related to indoor comfort feeling, and not to thermal sensation.

HF and LF/HF indicate a higher activation of the sympathetic nervous system under the indirect airflow condition during the execution of some tasks. Under the indirect airflow condition, HF showed lower responses during the CA and Cal sessions, and LF/HF also showed lower responses during the CA session. From the HF responses, we conclude that indirect airflow inhibited parasympathetic activity during task engagement in the CA and Cal sessions. Furthermore, the LF/HF responses indicated sympathetic nerve predominance under indirect airflow during the CA session. The differences in sympathetic/parasympathetic activity were not observed in the rest session but in the tasks to control external and internal attention. The results from the ECG measurements support that indirect airflow will induce a higher comfort in performing something that demands attention. These results can add different advantages from the EEG results that reflected comfort levels during relaxing.

In our experiments, the wind direction was changed as an independent variable, but the room temperature varied depending on the airflow condition. Therefore, we could not conclude that the observed responses were induced only by airflow sensation. Generally, it is very difficult to maintain the room temperature under different airflow directions in offices or schools using a commercial air-conditioner. Our experimental environment was closer to such general scenarios. However, more restricted controls will be necessary to reveal the physiological mechanism of airflow sensation in detail. Regarding the EEG analysis, we did not focus on other frequency bands like theta and alpha. Theta and other lower frequency bands can be influenced by various external noises; hence, we did not target lower frequency bands in the data measured near the indoor unit of the air-conditioner. For the alpha band, we previously reported that the amplitudes did not show differences under the different airflow environments for cooling [17]. In the future, another cognitive task that inhibits noise contamination tightly will enable to detect reliable activity in lower frequency bands like theta and delta. Finally, it has been reported that preferences of air movements differ between cooling and heating [9]. Future studies should investigate the responses under a heating environment with the same experimental procedure.

## 5 Conclusions

This study investigated the comfort of airflow direction by subjective and objective measurements, including face temperature, EEG, and ECG. The subjective assessments showed a relatively higher thermal sensation and pleasantness under indirect airflow. The mean face temperature was maintained under the indirect airflow conditions, but significantly decreased under direct airflow. The face-temperature changes supported the difference of pleasantness ratings between the airflow conditions. The longer psychological time under indirect airflow indicates low stress levels. The EEG and ECG responses indicated the different airflow effects in each task. The gamma and beta EEG were inhibited under indirect airflow, and the amplitudes negatively correlated with subjective assessments like pleasantness and thermal sensation during the rest session. The EEG results suggested a higher airflow comfort under the indirect airflow condition for the resting state. The ECG reactions indicated a predominant sympathetic activity during the CA and Cal sessions. Thus, indirect airflow can contribute to a better producibility in an indoor environment. These results provide reliable evidence to evaluate the comfort of an indirect airflow. In this study, we suggest a higher comfort under indirect airflow to faces from several aspects. Our procedures and results emphasize the effectiveness of combined subjective and objective measurements to reveal various aspects of airflow comfort.

## Supporting information

S1 Fig Gamma amplitudes during a) CA and b) Cal. The right row shows the topographies of the mean difference between indirect and direct airflow. White letters and circles indicate the maximum and minimum electrodes of the subtracted amplitudes. The color bar shows subtracted amplitudes (μV). The middle row shows the mean and standard errors of the amplitudes at the maximum electrodes. The X-axis shows the repetition, and the Y-axis shows the μV of the EEG amplitudes. The left row shows the mean and standard errors at the minimum electrodes.
(DOCX)

S2 Fig Beta amplitudes during a) CA, b) CM, and c) Cal. The right row shows the topographies of the mean difference between indirect and direct airflow. White letters and circles indicate the maximum and minimum electrodes of the subtracted amplitudes. The color bar shows subtracted amplitudes (μV). The middle row shows the mean and standard errors of the amplitudes at the maximum electrodes. The X-axis shows the repetition, and the Y-axis shows the μV of the EEG amplitudes. The left row shows the mean and standard errors at the minimum electrodes.
(DOCX)

S3 Fig ECG parameters of HF and LF/HF during a) Rest and b) CM. The right row shows the mean and standard error of HF, and the left row shows that of LF/HF. The X-axis shows the repetition.
(DOCX)

S4 Fig Scatter plots of each subjective assessment and gamma amplitudes at F7 during rest. Gray lines show the 95% density eclipse. Each point indicates individual mean data across three repetitions. There was no significant correlation (*P* > 0.05).
(DOCX)

S5 Fig Scatter plots of each subjective assessment and beta amplitudes at F7 during rest. Gray lines show the 95% density eclipse. Each point indicates individual mean data across three repetitions. There was no significant correlation (*P* > 0.05).
(DOCX)
