# Supplementary material for: Physiological and subjective comfort evaluation under different airflow directions in a cooling environment
Source: PLoS One. 2021 Apr 14;16(4):e0249235. doi: 10.1371/journal.pone.0249235 (PMC8046250; doi:10.1371/journal.pone.0249235)
Supplement: S2 Fig — Beta amplitudes during a) CA, b) CM, and c) Cal. The right row shows the topographies of the mean difference between indirect and direct airflow. White letters and circles indicate the maximum and minimum electrodes of the subtracted amplitudes. The color bar shows subtracted amplitudes (μV). The middle row shows the mean and standard errors of the amplitudes at the maximum electrodes. The X-axis shows the repetition, and the Y-axis shows the μV of the EEG amplitudes. The left row shows the mean and standard errors at the minimum electrodes. (DOCX) [file pone.0249235.s002.docx]

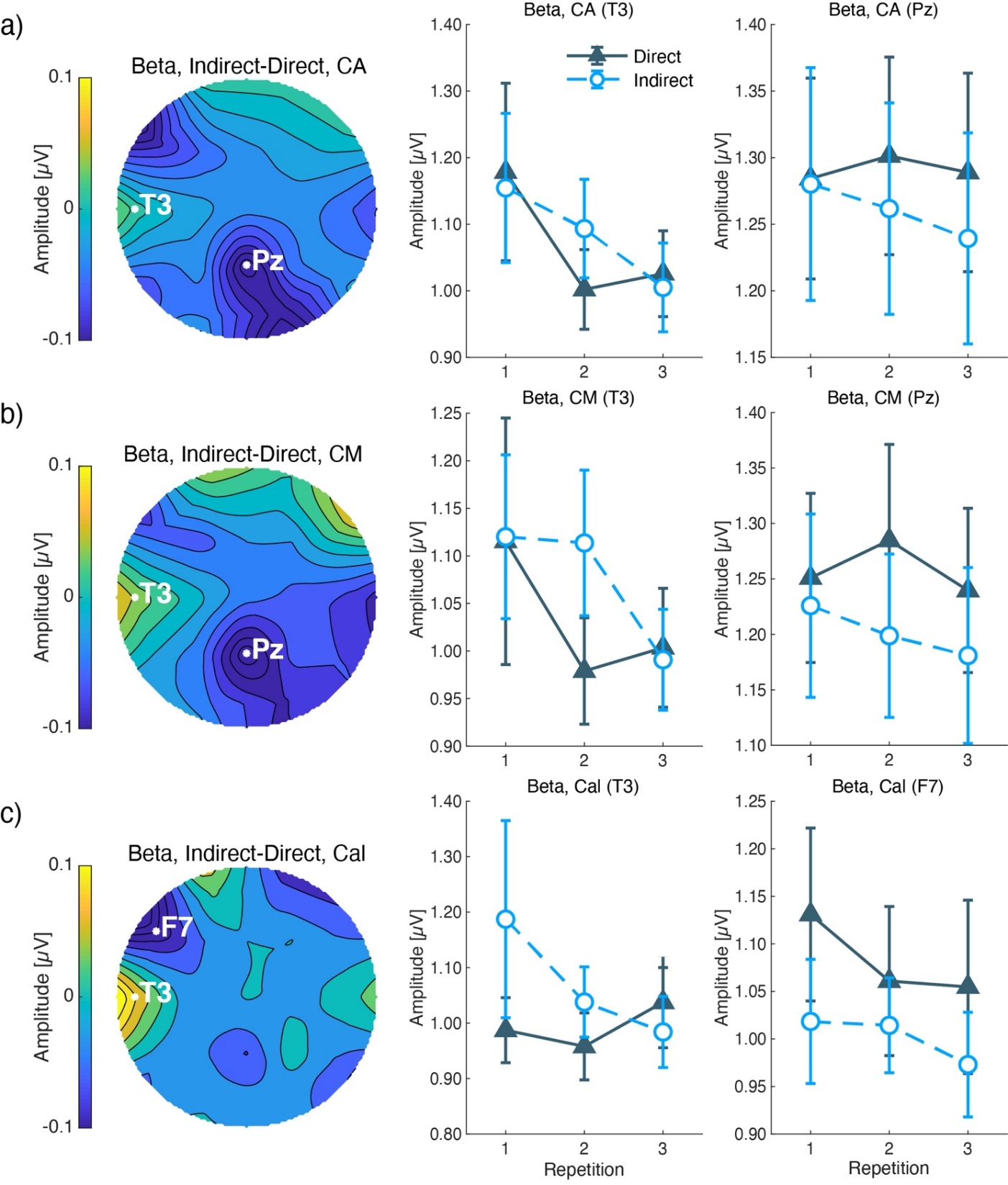


**Supplementary Figure 2.** Beta amplitudes during a) CA, b) CM, and c) Cal. The right row shows the topographies of the mean difference between indirect and direct airflow. White letters and circles indicate the maximum and minimum electrodes of the subtracted amplitudes. The color bar shows subtracted amplitudes (µV). The middle row shows the mean and standard errors of the amplitudes at the maximum electrodes. The X-axis shows the repetition, and the Y-axis shows the µV of the EEG amplitudes. The left row shows the mean and standard errors at the minimum electrodes.
