# Supplementary material for: Physiological and subjective comfort evaluation under different airflow directions in a cooling environment
Source: PLoS One. 2021 Apr 14;16(4):e0249235. doi: 10.1371/journal.pone.0249235 (PMC8046250; doi:10.1371/journal.pone.0249235)
Supplement: S3 Fig — ECG parameters of HF and LF/HF during a) Rest and b) CM. The right row shows the mean and standard error of HF, and the left row shows that of LF/HF. The X-axis shows the repetition. (DOCX) [file pone.0249235.s003.docx]

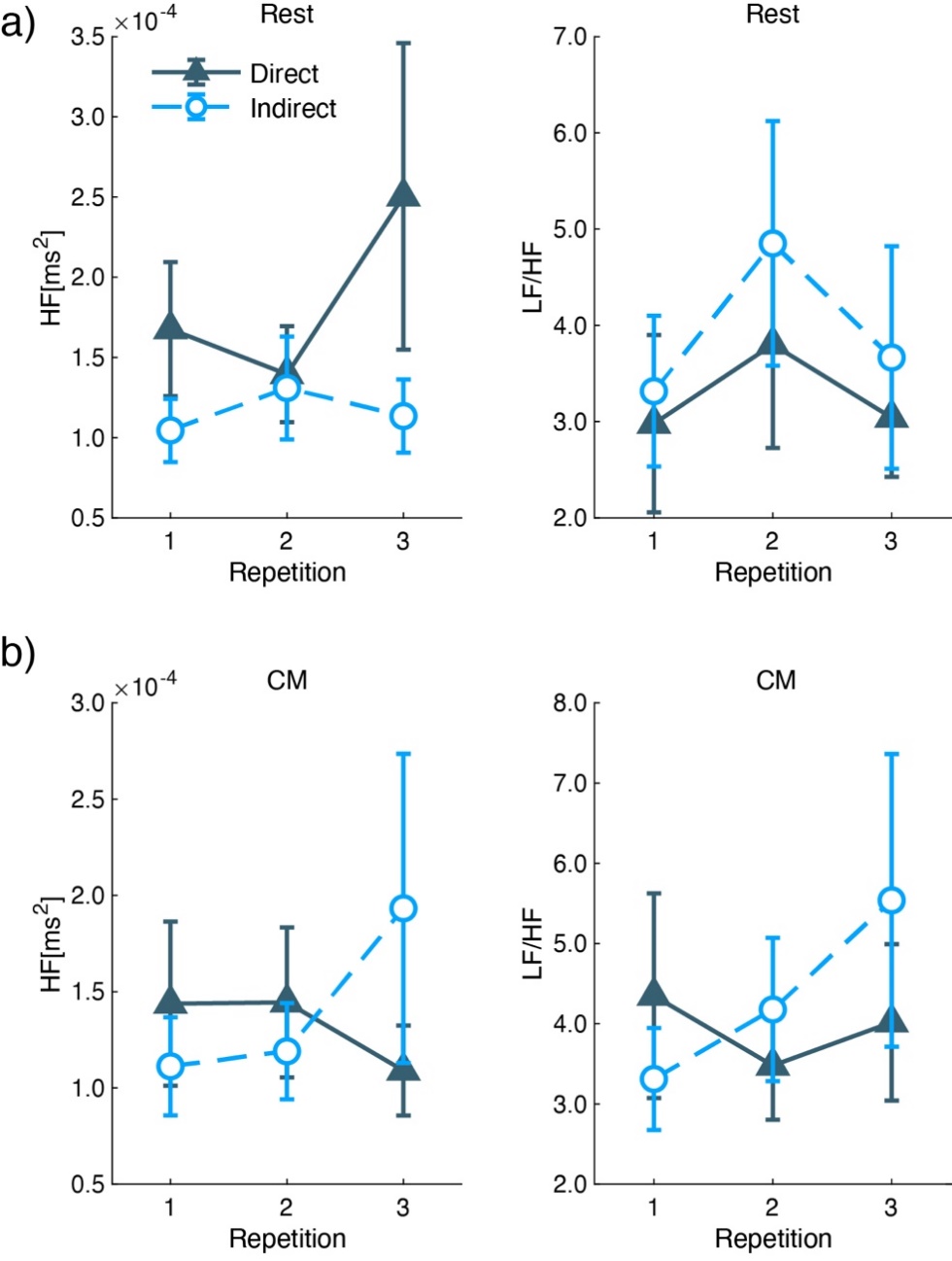


**Supplementary Figure 3.** ECG parameters of HF and LF/HF during a) Rest and b) CM. The right row shows the mean and standard error of HF, and the left row shows that of LF/HF. The X-axis shows the repetition.
