# Supplementary material for: Physiological and subjective comfort evaluation under different airflow directions in a cooling environment
Source: PLoS One. 2021 Apr 14;16(4):e0249235. doi: 10.1371/journal.pone.0249235 (PMC8046250; doi:10.1371/journal.pone.0249235)
Supplement: S4 Fig — Gray lines show the 95% density eclipse. Each point indicates individual mean data across three repetitions. There was no significant correlation (P > 0.05). (DOCX) [file pone.0249235.s004.docx]

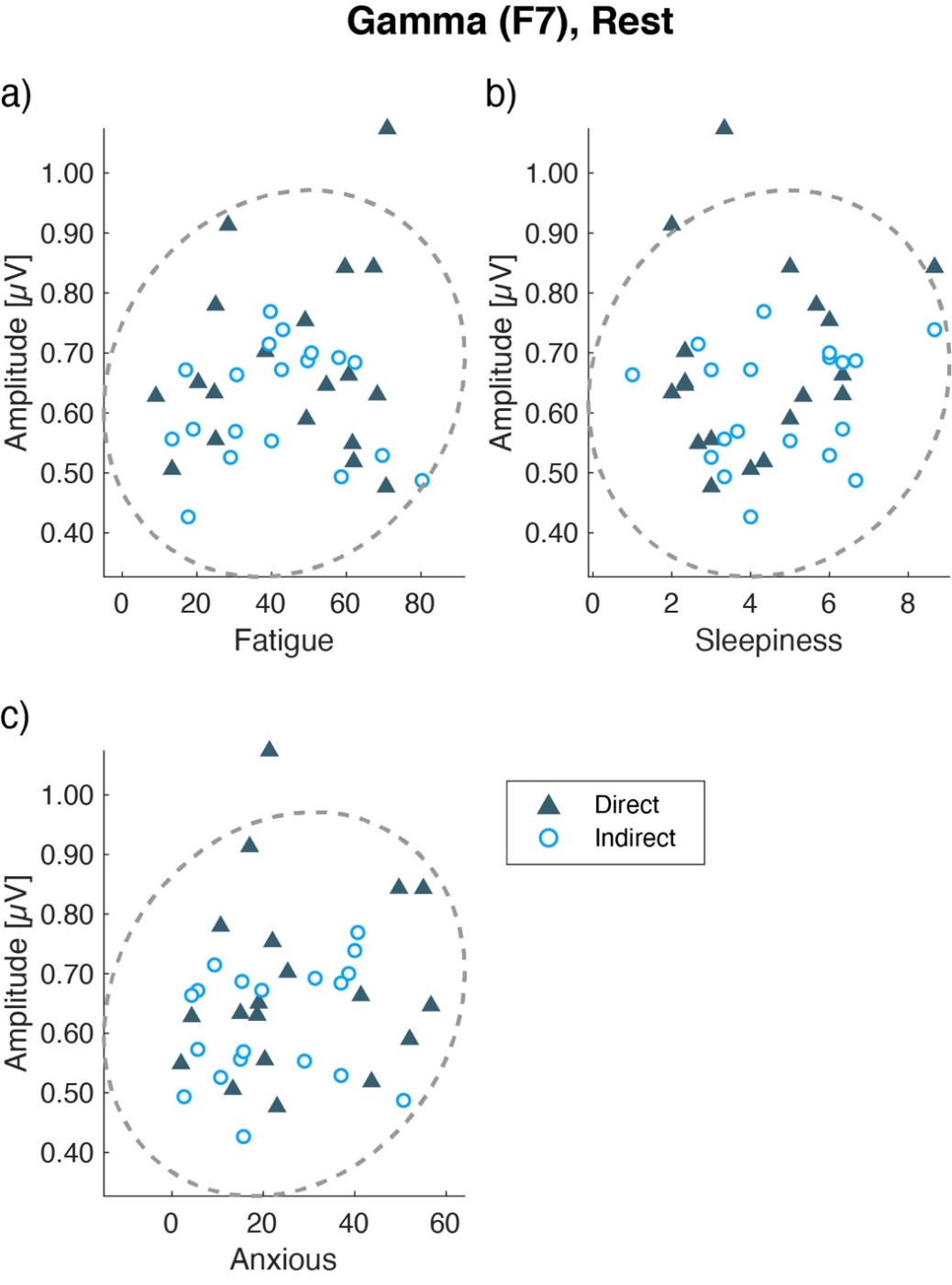


**Supplementary Figure 4.** Scatter plots of each subjective assessment and gamma amplitudes at F7 during Rest. Gray lines show the 95% density eclipse. Each point indicates individual mean data across three repetitions. There was no significant correlation (*P* > 0.05).
