# Supplementary material for: Physiological and subjective comfort evaluation under different airflow directions in a cooling environment
Source: PLoS One. 2021 Apr 14;16(4):e0249235. doi: 10.1371/journal.pone.0249235 (PMC8046250; doi:10.1371/journal.pone.0249235)
Supplement: Attachment — Submitted filename: response_letter2.1.docx [file pone.0249235.s006.docx]

**Decision letter from editor**

PONE-D-20-20091

Physiological and subjective comfort evaluation under different airflow directions in a cooling environment

PLOS ONE

Dear Dr. Okamoto,

Thank you for submitting your manuscript to PLOS ONE. After careful consideration, we have decided that your manuscript does not meet our criteria for publication and must therefore be rejected.

Specifically:

Editorial Comment: Two reviewers commented on your manuscript, and their suggestions can be found at the bottom of this letter. Particularly reviewer 1 found your work important but at the same time raised some methodological issues that would prevent publication in its present form. Since it is unclear whether you can handle these issues in a revision and in order to avoid long reviewer rounds, I decided to reject you paper for the moment. Having said that, I would be willing to accept a new re-submission of your paper should you believe you could deal with these comments in some way. In this case, I would expect you to prepare a revision of your work that addresses all concerns together with a cover letter that provides point-by-point replies. My own comments are provided below and are aimed to aid you in the process of revision.

I am sorry that we cannot be more positive on this occasion, but hope that you appreciate the reasons for this decision.

Yours sincerely,

Michael B. Steinborn, PhD

Academic Editor

PLOS ONE

**Response letter to editor and reviewers**

**To editor**

Additional Editor Comments:

1. Comment: measurement of airspeed
*Reviewer 1 raised the issue that airspeed is not correctly implemented. I am not the expert in this field of research, and I cannot judge whether this will compromise your results, however, the arguments raised by the reviewer seem convincing and should be addressed in a potential revision.*

Reply: We performed another experiment to measure wind direction and speed in multiple positions. The results obtained from the new experiment were added in Fig. 2–6. These results indicate that our experiment conditions were implemented properly.

2. Comment: design and counterbalancing
*Please specify how the conditions were arranged and what measures were taken to handle order effects. Personally, I think this point is important but at the same time, I would accept that a perfect design is oftentimes not possible due to practical constraints. At least, I would expect you to provide more details and justification of the present design technology.*

Reply: Our present design was counterbalanced and all participants performed both conditions in a different order. Half of the participants experienced the direct condition at first, and the other half experienced the indirect before the direct conditions.

3. Comment: test measures and reliability
*The time and counting tasks need more explanation with regard to implementation (number of trials, outlier treatment, computation of performance measures, etc.) and reliability. I suggest consulting my own methodological work which provides a tutorial of how to implement these measures (doi:10.1037/pas0000482; doi:10.1016/j.actpsy.2008.08.005) in basic and applied research settings.*

Reply: Thank you for your comments. According to the references, we added the number of trials, mean of the speeds, and split-half reliability coefficient in Table 4. We described the reliability in the Results as below:

“The mean durations of the 10-s counts in the CA, CM, and the difference of CM and CA are summarized in Table 4. The psychological time calculated by subtraction of CA’s duration from CM’s duration can be used as indicator of the relative changes of performance speeds. The reliability coefficients of psychological time were 0.934 and 0.777 under the direct and indirect condition, respectively (Table 4) [23,24].”

4. Comment: acclimatisation
*Reviewer 1 noted that some time is necessary for acclimatisation for the participants. I suggest elaborating on this point, and, if necessary, providing sufficient details in the revised manuscript.*

Reply: We started the measurement 20 min after the participants entered the experimental room. Then, they performed the task sequence for 20 min under one condition. That is, the participants were in the hot room at 32 °C for 20 min, then entered the experimental room (at 20 °C), stayed there for 20 min, and then started the experiment, which lasted for 20 min under each condition. We edited Fig. 1 and the Methods section to describe our design and acclimatization as below:

“To simulate the outside temperature, during the summer in Japan, each participant was asked to stay in a waiting room with the temperature set to 32 °C for 20 min. This acclimation to hot temperature counteracted the effect of variations in outside temperature during the experiment period. The participant then moved to the experimental room, where the room temperature was set to 20 °C. 20 min after the participant had entered the experimental room, physiological measurements and the experiment were started (Fig. 1d). The order of airflow conditions was counterbalanced for the participants. The air-conditioner was controlled before each measurement of a condition. Tasks the participants had to fulfill during the experiment consisted of four sessions: rest, time counting following alerts (CA), time counting in mind (CM), and mental calculation (Cal). This sequence was repeated three times for the direct and indirect airflow conditions in a random order (Fig. 1d). The sequence for one airflow condition took around 20 min. After one sequence, including three sessions, was finished, the next sequence started immediately.”

5. Comment: presenting results
*I suggest presenting statistical information in tables (if viable) and use economic ways of presenting figures. As Reviewer 1 suggested, scaling is important as it is not possible otherwise to understand the information presented in the figures.*

Reply: We revised the number of figures. The main figures were retained and others were added as supplementary data.

**Comments of reviewer #1**

*Comment: In the present study, the authors report measuring the effect of direction of airflow on multiple different measures. Unfortunately, I do not think that is what was actually measured and I have multiple major concerns about the study design. Such as:*

*・The authors cannot say they measured the effect of air direction, as the airspeed was not matched between trials. Therefore, what was actually measured was the effect of airspeed, altered by having the airflow coming from different directions.
・However, I also do not believe airspeeds were actually measured. The reported airspeeds are exceptionally low. I believe this stems from measurement error, where, from the authors diagram, air flow was coming from up above, but the anemometer was front-facing, therefore not catching what the actual airflow was.*

Reply: We performed another experiment to measure air direction and airspeed in multiple positions (Fig. 2–6). These results indicated that the airflow was blown from the front direction, and its speed was faster near the face of the participants (Tables 1 and 2, Figs. 3 and 5 at the Participant site, 60 cm height). Our results were obtained from the different airflow direction and speed effects.

*Comment: ・Both trials were conducted within the same lab-visit, back-to-back, and seemingly in rapid fire succession.
The authors take multiple measurements (thermal comfort and related measures) which are highly susceptible to changes in skin temperature. Skin temperature can take 15-30 minutes to reach a steady state in the temperature changes the participants undergo, going from the 30C room to the 20C room. Each airflow condition took ~12 minutes to complete, meaning skin temperature likely never reached a steady state. This is further complicated by the fact that the participants were likely lightly sweating when they came in from the 30C room, so being immediately confronted with airflow would of course make them highly uncomfortable.*

Reply: In the previous version of the manuscript the experimental design was not described in detail, including the acclimation time and task durations. The measurements started 20 min after the participants had entered the experimental room from the hot room. Each airflow condition took around 20 min to complete. We edited the method section to explain these procedures as below:

“To simulate the outside temperature, during the summer in Japan, each participant was asked to stay in a waiting room with the temperature set to 32 °C for 20 min. This acclimation to hot temperature counteracted the effect of variations in outside temperature during the experiment period. The participant then moved to the experimental room, where the room temperature was set to 20 °C. 20 min after the participant had entered the experimental room, physiological measurements and the experiment were started (Fig. 1d). The order of airflow conditions was counterbalanced for the participants. The air-conditioner was controlled before each measurement of a condition. Tasks the participants had to fulfill during the experiment consisted of four sessions: rest, time counting following alerts (CA), time counting in mind (CM), and mental calculation (Cal). This sequence was repeated three times for the direct and indirect airflow conditions in a random order (Fig. 1d). The sequence for one airflow condition took around 20 min. After one sequence, including three sessions, was finished, the next sequence started immediately.”

*Comment: P 3, L39: Is it direction or actually local air velocity (and subsequently local heat transfer and local change in temperature)*

Reply: We measured multiple local air directions and velocities as shown in Fig. 3–6. *Methods*

*Comment: L85: How was the required sample size determined?*

Reply: The sample size was determined according to the EEG study standard. The sample number determination corresponded with phase 1 of clinical trials in Japan, which is defined as 10–20 persons involved in assessing the safety of medical intervention.

*Comment: L85-86: I am happy to see a relative balance of male and female participants.
L93-94: Were all within-participant trials conducted during the same time period or were some participants tested over both periods?*

Reply: All within-participant trials were conducted during the same period. That is, every participant participated in either period, from 21 January 2019 to 1 February 2019 and from 10 May 2019 to 23 May 2019.

*Comment: L119-120: What kind of anemometer was it (please provide make and model)? How does it work? Was it stationary and front facing the entire time? Most anemometers that I am familiar with can only measure air velocity in a single plane. If the anemometer was front facing the entire time, I question whether it was measuring the actual air-flows as in your diagram, the air is coming from up above and not face on. Related, your wind velocities seem exceptionally low to me which makes me further question the set up of the anemometer.*

Reply: The PMV-meter (AM-101, Kyoto Electronics Manufacturing Co. LTD., Japan) used for physiological measurements was placed on the left side, as shown in Fig. 1b. The PMV-meter faced the same direction as that faced by the participants. For the measurement of airflow direction and velocity, we used a multi-channel anemometer (Multi-channel anemometer 1550 series, KANOMAX JAPAN INC. Osaka, Japan) and probe cables (MODEL 1504 probe cable velocity channel, KANOMAX JAPAN INC. Osaka, Japan). The results with every plane are shown in Fig.3–6.

*Comment: L122: Why were hospital gowns selected instead of a standardized set of clothing that would have similar clothing insulation values as everyday clothing? Unless this was specific to hospital environments, in which case that should be mentioned in the introduction.*

Reply: We chose disposable and thin gowns for the experiments not specific to hospital. The fabric of the gown was so thin that the participants could feel changes in room temperature sensitively.

*Comment: L123: why was blood oxygen saturation measured?*

Reply: Blood oxygen saturation was measured for the confirmation of the safety of the environment. The data were not related to the main result.

We now describe the rationale for measuring blood oxygen saturation as below:

“The blood oxygen saturation (SpO2) was measured by a finger pulse oximeter to confirm safe oxygen levels of more than 95%. (Fig. 1b). When we observed oxygen levels higher than the safety levels during the experiments, the results of SpO2 were not reported in the Results section.”

*Comment: L113-126: What was the total duration that the participants were in the 20C room for?
Similar, how long were the participants in the 20C room for before measurements began?
It can take 15-30 minutes for skin temperature to stabilize when changing rooms with that much of a temperature difference.*

Reply: Similar to our response to a previous comment above, physiological measurements started 20 min after the participants entered the 20 °C room. For one condition, the experimental sequence took 20 min. The total duration the participants stayed in the experimental room was about 60 min.

*Comment: L127: What tasks?*

Reply: In each session, different tasks were instructed to be performed: a time counting task in CA and CM sessions, and mental calculation in Cal. We describe each task in the method section as below:

“The CA and CM sessions were designed to measure psychological time. In the CA session, each participant was required to press a button following time alerts every 10 s for 60 s. That is, they pressed the button six times in a session. In the CM session, the participants were asked to estimate 10 s as accurately as possible and press the button after counting silently for 10 s; this task was repeated six times (~ 60 s). The measured psychological time in the CA and CM sessions were analyzed as described in section 2.6.”

“In the Cal session, each participant was asked to consecutively subtract 13 from a four-digit number (e.g., 1012−13 = 999, 999−13=…) as quickly as possible for 60 s. The four-digit number was randomly assigned, ranging from 1000 to 1012 for each participant.”

*Comment: L153-158: Again, you are saying before and after the measurement, but I do not know how long they were exposed to the air for.
Also, according to your figure 1d, it appears as though you took the thermography measurement, ran the first air speed condition, then ran the second condition, then took a follow up thermography measurement. How do you have a pre and a post for both air speed conditions?*

Reply: Similar to one of our responses to a previous comment, one condition lasted for 20 min. That is, the exposure time for the air was about 20 min, and the thermograph data were measured pre and post the exposures. We edited Fig. 1d to explain the detailed procedure and timing when the thermography data were measured.

*Results*

*Comment: Why SEM instead of SD?*

Reply: In our manuscript, we performed parametric statistics to test the difference of mean values, and SEM indicates how much discrepancy there is likely to be in a sample's mean compared to the true population mean. On the other hand, SD indicates how accurately the mean reflects the sample data. Parametric statistics estimate the mean value of the true population mean from the sample data, and SEM is used to compute significance and confidence intervals for the mean difference. Thus, SEM corresponded with parametric statistics for hypothesis testing of the mean difference.

*Comment: Did the authors test for a trial-order effect? Based on the short transition time between the 30C room and the 20C room, I would imagine scores were much higher for whatever air speed condition was tested first.*

Reply: The participants stayed 20 min after they entered the 20 °C room. The air conditions were counterbalanced so that it was not necessary to test order effects of the air conditions. In each condition, we considered repetition effects using repeated-measures ANOVA.

*Comment: Throughout, report the same number of decimal places for mean and error.*

Reply: All of the decimals were rounded to three decimal places.

*Discussion*

*Comment: You can't fairly conclude that participants preferred indirect air flow with such short trial durations. Especially since they were probably sweating from the 30C room, so airflow would feel terrible as that sweat evaporates.*

Reply: In the first paragraph of the discussion, we edited the expressions as below:

“In this study, we aimed to evaluate the effects of the environment, which were induced by different airflow directions, on indoor comfort with subjective assessments and physiological measurements. As expected, we confirmed that the subjective assessments and some physical measurements indicate higher pleasantness under the environment with indirect airflow. ”

For the explanation of the subjective assessments, we also edited the key sentence as below:

“The subjective assessments indicate that the indoor environment with indirect airflow was more pleasant than that with direct airflow.”

In the limitation paragraph of the discussion, we discussed this problem as below:

“In our experiments, the wind direction was changed as an independent variable, but the room temperature varied depending on the airflow condition. Therefore, we could not conclude that the observed responses were induced only by airflow sensation.”

*Figures*

*Comment: For your perceptual measures consider adding qualitative values so the readers know what the scores mean immediately when looking at the figures. What is feeling 30 anxious?*

We edited the figure for perceptual measures. Qualitative values were added to the figure. Please refer to Fig. 8.

**To reviewer #2**

*Comment: 1. Line 114 and 115, are there some references for acclimation for 20 min?*

Reply: As reviewer 1 pointed out in the comment on L113-126, it can take 15-30 minutes for the skin temperature to stabilize after the room temperature has changed. In this case, the experimental sequence for one condition lasted for approximately 20 min, so that the duration of acclimation was set to 20 min.

*Comment: 2. Line 117, why was 20 oC selected? In summer, indoor air temperature is usually set as 25 oC.*

Reply: To focus on the airflow effect, we chose a room temperature that can arouse differences in physiological activity. Especially in neuronal responses, the signals are stable and robust toward environmental changes. To detect characteristics from physiological activities, a somewhat excessive setting, which would influence the participants’ neuronal signals, was needed.

*Comment: 3. As far as I know, LF/HF is easily affected by individual differences. How do authors avoid such error?*

Reply: We did not remove any outlier data points caused by individual differences. For the analysis of R-R intervals, we conducted two-way ANOVA for within-subject analysis, which can identify the variability that is uniquely caused by individual differences. The analysis can show the difference LF/HF of the two airflow conditions, despite retaining individual errors. We have analyzed CVRR for the parameter of R-R intervals, but there was no significant effect (data not shown). We cannot identify if the CVRR result was affected by individual differences or not.

*Comment: 4. What time were experiments tested in one day? Thermal response is a little different between morning and afternoon because of circadian rhythm.*

Reply: We are aware of the relationship between thermal responses and the circadian rhythm, but it was difficult to gather enough samples in the given experimental time on one day and with limited access to the experimental room. The participants took part in the experiments at any of the following four time slots: 9:00–11:00, 11:00–13:00, 13:00–15:00, 15:00–17:00.

*Comment: 5. All the figures are not clear.*

Reply: We revised the figure number and order. Only the main results were retained, and the other figures were moved to the supplementary figures.

*Comment: 6. In Fig. 2 (a), letter "p" is not clear in "repetition".*

Reply: We corrected the figure, which is now the new Fig. 7.
