# Supplementary material for: Physiological and subjective comfort evaluation under different airflow directions in a cooling environment
Source: PLoS One. 2021 Apr 14;16(4):e0249235. doi: 10.1371/journal.pone.0249235 (PMC8046250; doi:10.1371/journal.pone.0249235)
Supplement: Peer review history

# Author comment: Author response to previous submission

**DOI:** 10.1371/journal.pone.0249235.r001

8 Mar 2021

Attachment Submitted filename: response_letter2.1.docx Click here for additional data file.

# Editor report: Decision Letter 0

**DOI:** 10.1371/journal.pone.0249235.r002

**Authors:** Michael B. Steinborn

15 Mar 2021

Physiological and subjective comfort evaluation under different airflow directions in a cooling environment

PONE-D-21-07568

Dear Dr. Okamoto,

We’re pleased to inform you that your manuscript has been judged scientifically suitable for publication and will be formally accepted for publication once it meets all outstanding technical requirements.

Within one week, you’ll receive an e-mail detailing the required amendments. When these have been addressed, you’ll receive a formal acceptance letter and your manuscript will be scheduled for publication.

An invoice for payment will follow shortly after the formal acceptance. To ensure an efficient process, please log into Editorial Manager at http://www.editorialmanager.com/pone/, click the 'Update My Information' link at the top of the page, and double check that your user information is up-to-date. If you have any billing related questions, please contact our Author Billing department directly at authorbilling@plos.org.

If your institution or institutions have a press office, please notify them about your upcoming paper to help maximize its impact. If they’ll be preparing press materials, please inform our press team as soon as possible -- no later than 48 hours after receiving the formal acceptance. Your manuscript will remain under strict press embargo until 2 pm Eastern Time on the date of publication. For more information, please contact onepress@plos.org.

Kind regards,

Michael B. Steinborn, PhD

Academic Editor

PLOS ONE

Additional Editor Comments:

I have read the revised manuscript with pleasure. The way and the detail by which you handled each of the reviewer's comments is appreciable. The manuscript itself is insightful written and well evaluated. The results are interesting and well communicated. The results are supported by a variety of performance data, which is also a strength of the manuscript. Remaining weak points are addressed properly. 

# Editor report: Acceptance letter

**DOI:** 10.1371/journal.pone.0249235.r003

**Authors:** Michael B. Steinborn

23 Mar 2021

PONE-D-21-07568 

Physiological and subjective comfort evaluation under different airflow directions in a cooling environment 

Dear Dr. Okamoto:

I'm pleased to inform you that your manuscript has been deemed suitable for publication in PLOS ONE. Congratulations! Your manuscript is now with our production department. 

If your institution or institutions have a press office, please let them know about your upcoming paper now to help maximize its impact. If they'll be preparing press materials, please inform our press team within the next 48 hours. Your manuscript will remain under strict press embargo until 2 pm Eastern Time on the date of publication. For more information please contact onepress@plos.org.

If we can help with anything else, please email us at plosone@plos.org. 

Thank you for submitting your work to PLOS ONE and supporting open access. 

Kind regards, 

PLOS ONE Editorial Office Staff

on behalf of

Dr. Michael B. Steinborn 

Academic Editor

PLOS ONE
